# Realization of vertical metal semiconductor heterostructures via solution phase epitaxy

**DOI:** 10.1038/s41467-018-06053-z

**Published:** 2018-09-06

**Authors:** Xiaoshan Wang, Zhiwei Wang, Jindong Zhang, Xiang Wang, Zhipeng Zhang, Jialiang Wang, Zhaohua Zhu, Zhuoyao Li, Yao Liu, Xuefeng Hu, Junwen Qiu, Guohua Hu, Bo Chen, Ning Wang, Qiyuan He, Junze Chen, Jiaxu Yan, Wei Zhang, Tawfique Hasan, Shaozhou Li, Hai Li, Hua Zhang, Qiang Wang, Xiao Huang, Wei Huang

**Affiliations:** 1 Institute of Advanced Materials (IAM), Nanjing Tech University (NanjingTech), 30 South Puzhu Road, Nanjing, 211816 China; 2 State Key Laboratory of Materials-Oriented Chemical Engineering, College of Chemical Engineering, Nanjing Tech University (NanjingTech), 30 South Puzhu Road, Nanjing, 211816 China; 3 Cambridge Graphene Centre, University of Cambridge, Cambridge, CB3 0FA UK; 4 Center for Programmable Materials, School of Materials Science and Engineering, Nanyang Technological University, 50 Nanyang Avenue, Singapore, 639798 Singapore; 5 Key Laboratory for Organic Electronics and Information Displays & Institute of Advanced Materials, Jiangsu National Synergistic Innovation Center for Advanced Materials (SICAM), Nanjing University of Posts & Telecommunications, 9 Wenyuan Road, Nanjing, 210023 China; 6 School of Chemistry and Molecular Engineering, Nanjing Tech University (NanjingTech), 30 South Puzhu Road, Nanjing, 211816 China; 7 Shaanxi Institute of Flexible Electronics (SIFE), Northwestern Polytechnical University (NPU), 127 West Youyi Road, Xi’an, 710072 China

## Abstract

The creation of crystal phase heterostructures of transition metal chalcogenides, e.g., the 1T/2H heterostructures, has led to the formation of metal/semiconductor junctions with low potential barriers. Very differently, post-transition metal chalcogenides are semiconductors regardless of their phases. Herein, we report, based on experimental and simulation results, that alloying between 1T-SnS_2_ and 1T-WS_2_ induces a charge redistribution in Sn and W to realize metallic Sn_0.5_W_0.5_S_2_ nanosheets. These nanosheets are epitaxially deposited on surfaces of semiconducting SnS_2_ nanoplates to form vertical heterostructures. The ohmic-like contact formed at the Sn_0.5_W_0.5_S_2_/SnS_2_ heterointerface affords rapid transport of charge carriers, and allows for the fabrication of fast photodetectors. Such facile charge transfer, combined with a high surface affinity for acetone molecules, further enables their use as highly selective 100 ppb level acetone sensors. Our work suggests that combining compositional and structural control in solution-phase epitaxy holds promises for solution-processible thin-film optoelectronics and sensors.

## Abstract (web summary)

Controlling the composition and crystal phase of layered heterostructures is important. Here, the authors report the liquid-phase epitaxial growth of Sn_0.5_W_0.5_S_2_ nanosheets with 83% metallic phase on SnS_2_ nanoplates, which are used as 100 ppb level chemiresistive gas sensors at room temperature.

## Introduction

Heterostructures constructed from layered materials such as graphene, metal chalcogenides and black phosphorus (BP) have aroused particular interest due to their combined advantageous features and the emergence of unusual properties/functions^1–6^. As a group of the mostly studied layered materials, transition metal chalcogenides (TMCs) like MoS_2_ and WS_2_ exist in different crystal phases such as the 2H and 1T polytypes with distinct electronic properties. Their semiconductor-to-metal transition (i.e., 2H-to-1T) can be realized via Li-intercalation,^7^ strain engineering,^8^ e-beam/laser irradiation^2^ or doping/alloying^2,7–9^. This attractive feature allows for the formation of 2H/1T (semiconductor/metal) phase junctions that exhibit much reduced contact resistance compared to that of using noble metal contacts (e.g., Au) which normally interface with semiconducting TMCs with large Fermi level misalignment^2,10^. Besides TMCs, post-TMCs such as SnS_2_ and InSe are another important group of layered materials, exhibiting attractive electronic and optoelectronic properties for a wide range of applications, including transistors, photodetectors, and sensors^11,12^. Similar to many other semiconducting materials, the type of metal contact with post-TMCs plays a critical role in tuning their functional performance^13^. However, due to the less metallic nature of post-transition metals, post-TMCs are generally semiconductors regardless of their crystal phases, such as the 2H, 4H, and 1T polytypes^14^. Consequently, contacting post-TMCs with metallic layered materials to achieve low interfacial resistance remains a big challenge.

To date, much effort has been devoted to the preparation of various heterostructures based on layered materials, which display different geometric arrangements such as the lateral and vertical heterostructures^1,3,15^. In particular, to prepare vertical heterostructures in which dissimilar layered crystals are stacked one above the other in a pre-designed sequence, solid-state procedures, including dry transfer, chemical vapor deposition (CVD) and chemical vapor transport (CVT) method have mostly been applied^3,16,17^. This is because these methods allow good control over the spatial arrangement of the layers as well as the deposition sequence. Compared to these solid-state methods, solution-phased approaches are advantageous in terms of relatively easier procedures, low-cost setups, and most importantly, scalability^18,19^. However, direct wet-chemical growth of vertical heterostructures of layered metal chalcogenides has thus far been challenging.

In view of their potential applications, heterostructures/heterojunctions such as InSe/graphene, MoTe_2_/MoS_2_, MoS_2_/perovskite, and graphene/MoS_2_/graphene have recently shown promising performance in photodetectors, due to the improved charge separation/transport and enhanced light adsorption^20–23^. Meanwhile, development of gas sensors for detection of volatile organic compounds are important in applications such as environmental monitoring and non-invasive diagnosis of diseases based on breath analysis^24,25^. Chemiresistive sensors based on metal oxides/sulfides have been used for detection of volatile organic compounds, however, high operating temperatures (typically ≥150 °C) are usually required to achieve good sensitivity and selectivity^24,26^. Very recently, layered materials such as SnS_2_, WS_2_ and Ti_3_C_2_T_*x*_ have demonstrated great potential for room-temperature gas detection^27–29^. It is expected that creation of heterostructures may realize further improved sensing performance^30–32^.

In this contribution, nanoplates of SnS_2_, a typical n-type semiconductor, are used as synthesis templates for the surface deposition of layered Sn_0.5_W_0.5_S_2_ nanosheets, which show 83% metallic phase, leading to the formation of metal/semiconductor vertical heterostructures. Kelvin probe force microscope (KPFM) and tunneling atomic force microscopy (TUNA) analyses suggest the formation of ohmic-like contact at the Sn_0.5_W_0.5_S_2_/SnS_2_ interface. The resultant heterostructures are fabricated into chemiresistive sensors to detect acetone at room temperature and exhibit a good selectivity and a minimum detectable concentration down to 100 ppb. The good sensing performance could be attributed to the low charge transfer resistance at the Sn_0.5_W_0.5_S_2_/SnS_2_ interface that enables a much increased signal-to-noise ratio, and the alloying induced enhancement in surface gas adsorption.

## Results

### Synthesis and characterizations of Sn_0.5_W_0.5_S_2_/SnS_2_

**Fig. 1:**
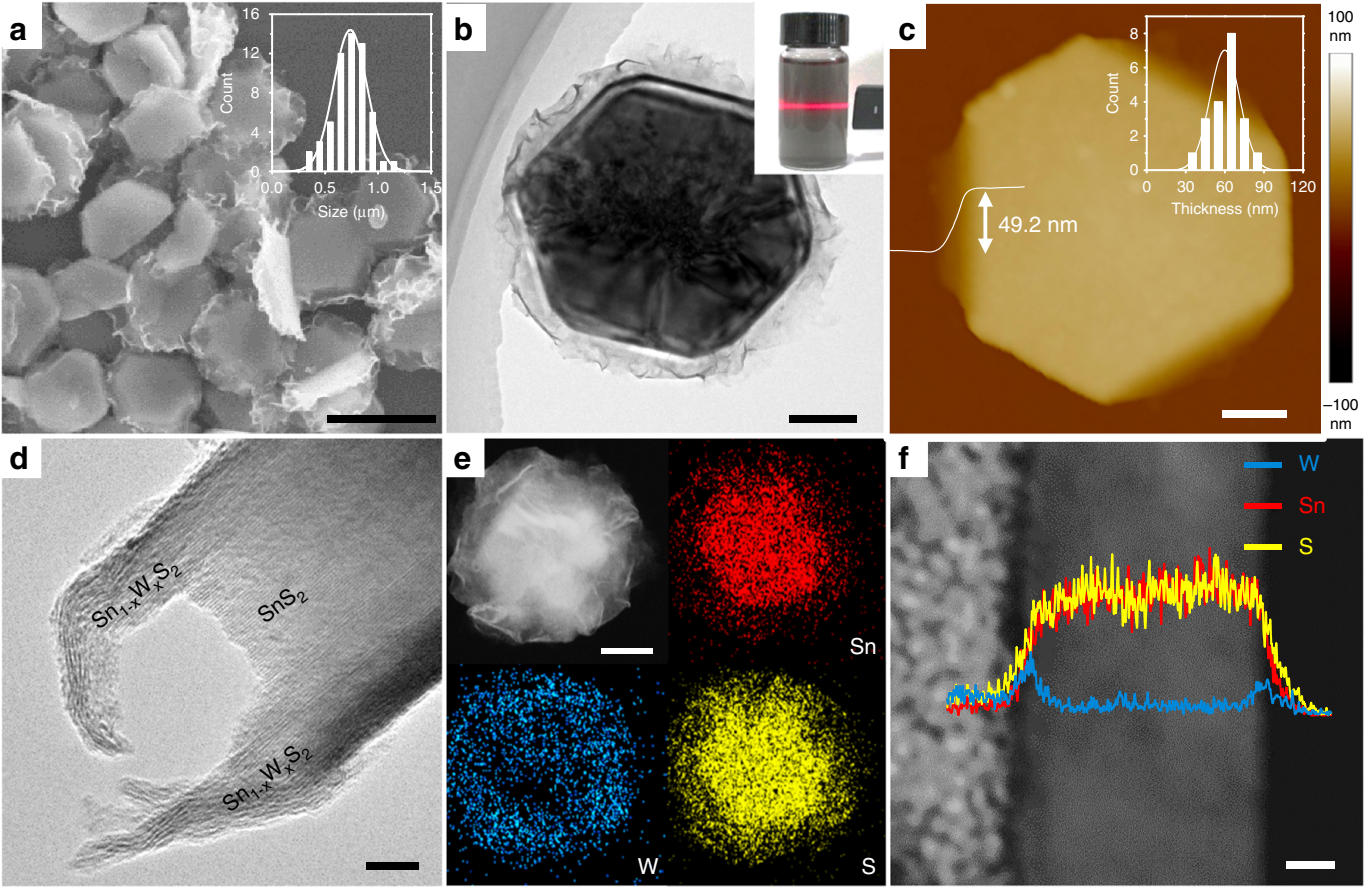
Morphology and composition analyses of Sn_0.5_W_0.5_S_2_/SnS_2_ heterostructures. **a** SEM image of as-prepared Sn_1–*x*_W_*x*_S_2_/SnS_2_ heterostructures (scale bar, 1 μm). Inset: size distribution of Sn_1–*x*_W_*x*_S_2_/SnS_2_ heterstructures. **b** Top-view TEM image of typical Sn_1–*x*_W_*x*_S_2_/SnS_2_ heterostructures (scale bar, 200 nm). Inset: photograph of a solution of Sn_1–*x*_W_*x*_S_2_/SnS_2_ heterostructures showing the Tyndall effect. **c** AFM image and height analysis of a Sn_1–*x*_W_*x*_S_2_/SnS_2_ heterostructure (scale bar, 100 nm). Inset: thickness distribution of Sn_1–*x*_W_*x*_S_2_/SnS_2_ heterstructures, showing a mean value of 60 nm. **d** Side-view TEM image of a typical Sn_1–*x*_W_*x*_S_2_/SnS_2_ heterostructure, revealing Sn_1–*x*_W_*x*_S_2_ nanosheets grown on both the top and bottom basal faces of a SnS_2_ nanoplate (scale bar, 10 nm). **e** STEM image and EDX mapping on a typical Sn_1–*x*_W_*x*_S_2_/SnS_2_ heterostructure (scale bar, 100 nm). **f** Cross-sectional STEM image and EDX line analysis on a typical Sn_1–*x*_W_*x*_S_2_/SnS_2_ heterostructure (scale bar, 10 nm)

As a representative post-TMC, SnS_2_ has been widely studied and applied in phototransistors and sensors for its favorable band structure and relatively high surface electronegativity^12,33^. Typically, SnS_2_ nanoplates were synthesized via a hydrothermal reaction with thiourea (CS(NH_2_)_2_) and tin tetrachloride hydrate (SnCl_4_•5H_**2**_O) as the precursors for S and Sn, respectively (see the Methods section for the detailed procedure)^34^. As shown in the scanning electron microscope (SEM), transmission electron microscope (TEM) and atomic force microscopy (AFM) images in Supplementary Figs. 1 and 2, hexagonal nanoplates with edge lengths ranging from 200 to 700 nm and an average thickness of 43 nm were obtained. As confirmed by the selected area electron diffraction (SAED) and X-ray diffraction (XRD) analyses, the nanoplates are α-SnS_2_ with a 1T structure (space group *P*$\bar 3$*m1*), where *a* = 3.65 and *c* = 5.90 (ICSD no. 42566)^12^ (Supplementary Figs. 1c and d). By adding (NH_4_)_10_H_2_(W_2_O_7_)_6_ to the precursors of the aforementioned synthesis solution, alloyed Sn_1–*x*_W_*x*_S_2_ nanosheets were in-situ synthesized and hybridized with SnS_2_ nanoplates as shown in Fig. 1a, b. These heterostructures show an average lateral size of 750 nm (Fig. 1a) and an average thickness of 60 nm (Fig. 1c and Supplementary Fig. 3). The side-view TEM image in Fig. 1d clearly shows a SnS_2_ nanoplate covered by Sn_1–*x*_W_*x*_S_2_ nanosheets on both its basal faces forming a vertical heterostructure. The deposited Sn_1–x_W_x_S_2_ nanosheets are typically 6–9 nm in thickness (Supplementary Fig. 4). Energy dispersive X-ray spectroscopy (EDX) mapping of a typical heterostructure reveals the distribution of Sn, W, and S elements (Fig. 1e), in which the center of the heterostructure shows a higher concentration of Sn compared to the edge. EDX line analysis of the cross-section of a typical heterostructure, which was prepared by cutting with focused ion beam (FIB), further indicates that the SnS_2_ nanoplate was covered by Sn_1–*x*_W_*x*_S_2_ nanosheets (Fig. 1f). EDX spot analyses on edges of Sn_1–*x*_W_*x*_S_2_ nanosheets suggest *x* ≈ 0.5, confirming Sn_0.5_W_0.5_S_2_ nanosheets were obtained (Supplementary Fig. 5).

**Fig. 2:**
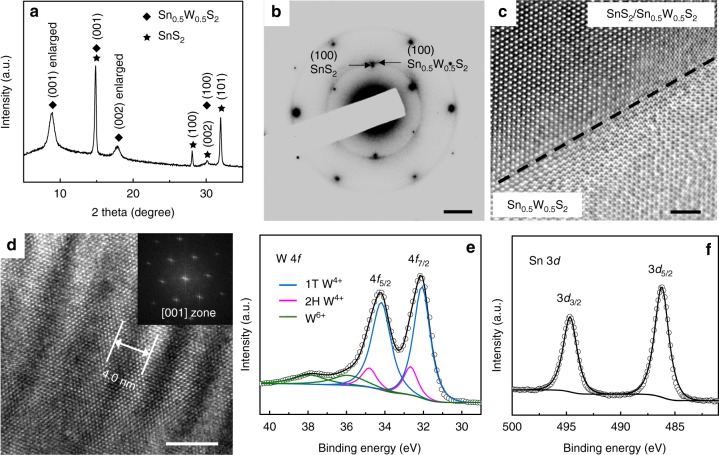
Structural properties of Sn_0.5_W_0.5_S_2_/SnS_2_ heterostructures. **a** XRD pattern of Sn_0.5_W_0.5_S_2_/SnS_2_ heterostructures deposited on a glass slide. **b** SAED patterns of a Sn_0.5_W_0.5_S_2_/SnS_2_ heterostructure along the [001] zone axis (scale bar, 2 nm^−1^). **c** HRTEM image of a typical Sn_0.5_W_0.5_S_2_/SnS_2_ heterostructure lying flatly on a copper grid (scale bar, 2 nm). **d** A Moiré pattern with a periodicity of 4.0 nm was observed (scale bar, 5 nm), whose fast Fourier transform (FFT) diffraction pattern is shown as the inset. XPS (**e**) W 4*f* and **f** Sn 3*d* spectra of as-prepared Sn_0.5_W_0.5_S_2_/SnS_2_ heterostructures

The structural properties of the heterostructures were investigated with XRD and high resolution TEM (HRTEM) analysis. In the XRD pattern in Fig. 2a, besides the characteristic peaks for 1T-SnS_2_, two relatively broader peaks observed at 8.9° and 17.8° could be attributed, respectively, to the (001) and (002) planes of Sn_0.5_W_0.5_S_2_ nanosheets with an enlarged interlayer spacing of about 1.0 nm. The enlarged spacing, which was also observed in side-view HRTEM images (Supplementary Fig. 6), may result from the use of CS(NH_2_)_2_ and (NH_4_)_10_H_2_(W_2_O_7_)_6_ in our synthesis, leading to the intercalation of NH_4_^+^ ions in between the adjacent layers^35^. This was also confirmed by X-ray photoelectron spectroscopy (XPS) analysis (Supplementary Fig. 7). The crystal structure of a typical heterostructure was further investigated by taking its SAED pattern. As shown in Fig. 2b, two sets of patterns both along the [001] zone axis show the epitaxial registry. The hexagonal pattern with six inner and six outer spots can be assigned to 1T-SnS_2_, and the measured (100) lattice spacing is 3.16 Å, in good agreement with the XRD result (Fig. 2a). The other set of pattern for Sn_0.5_W_0.5_S_2_ shows elongated spots, forming discontinued ring segments. This may result from the curled structure of the Sn_0.5_W_0.5_S_2_ nanosheets as well as possible misorientation. The measured lattice spacing for Sn_0.5_W_0.5_S_2_ (100) planes was 3.0 Å, corresponding to a lattice parameter of *a* = 3.46 Å. This reasonably falls in between that of WS_2_ (*a* = 3.16 Å) and SnS_2_ (*a* = 3.65 Å). Note that the mismatch between the (100) planes of SnS_2_ and Sn_0.5_W_0.5_S_2_ is 5%, which can be tolerated in van der Waals epitaxial growth of layered materials^36^. The top-view HRTEM image in Fig. 2c distinctly shows the relatively darker region for the SnS_2_ nanoplate covered with Sn_0.5_W_0.5_S_2_, and the brighter region for the periphery Sn_0.5_W_0.5_S_2_ nanosheet. The lattice fringes extend continuously from the SnS_2_/Sn_0.5_W_0.5_S_2_ center to the Sn_0.5_W_0.5_S_2_ edge, further confirming the epitaxial growth mode. Moiré patterns could be observed in some areas due to the overlap of Sn_0.5_W_0.5_S_2_ and SnS_2_ at small misorientation angles. For example, as shown in Fig. 2d, a Moiré pattern with a periodicity of 4.0 nm was observed when the Sn_0.5_W_0.5_S_2_ overlayer made a misorientation angle of 3° with SnS_2_ (see the detailed analysis in Supplementary Fig. 8). Note that the overlapping of two hexagonal lattice patterns normally produces a hexagonal Moiré pattern^37^, which was not observed in the present work. This may be due to the fact that the Sn_0.5_W_0.5_S_2_ nanosheets showed lattice distortion with varied interlayer spacings (0.6–1.0 nm, Supplementary Fig. 6), and thus were deviated from being perfectly flat on the SnS_2_ nanoplate. As a result, only short-range line-like Moiré patterns were observed^38^. High resolution scanning TEM (STEM) images of the edge area of a heterostructure show the 1T-phase-like atomic arrangement of Sn_0.5_W_0.5_S_2_ (Supplementary Fig. 9)^39^. It is interesting that zigzag lattice patterns for 1T’ or T_d_ phases which have been previously observed in TMCs such as WS_2_ and WTe_2_^40,41^ were not observed in Sn_0.5_W_0.5_S_2_. Based on our density functional theory (DFT) calculation results shown in Supplementary Table 1 and Supplementary Fig. 10, the W–S bonds tend to be shorter than the Sn–S bonds in the alloyed system, resulting in a distortion from the perfect in-plane symmetric 1T lattice. The distorted 1T structure was also reflected in the Raman spectrum in Supplementary Fig. 11, where in addition to the peaks at 310, 351, and 414 cm^−1^ that correspond to the SnS_2_-like *A*_1g_, WS_2_-like *E*_2g_ and WS_2_-like *A*_1g_ modes, respectively, the active modes observed at 171 cm^−1^ and 224 cm^−1^ in the lower frequency region could be attributed to distorted 1T-WS_2_^12,35^. The dominant distorted 1T-phased W–S coordination was further confirmed by XPS analysis (Fig. 2e). It has been reported that the XPS band positions of a metal element are sensitive to their oxidation states, coordination geometries, and Fermi levels^42,43^. Normally in the W*f* spectrum, the doublet peaks (32.1 and 34.2 eV) associated with W in the 1T-phased structure are downshifted by 0.6 eV relative to those associated with W in the 2H structure (32.7 and 34.8 eV). The deconvolution of the W 4*f* bands thus could enable the quantitative estimation of the 1T and 2H phase concentrations^7^. In our case, from the deconvoluted peak areas (Fig. 2e), the concentrations of the distorted 1T and 2H phases were calculated to be 83% and 17%, respectively. For the Sn 3*d* spectrum, the peak positions match well with the 1T-SnS_2_ structure (Fig. 2f)^44^.

The formation of 1T or distorted 1T structures have been observed previously in TMCs when they were intercalated with alkali metal ions (e.g., Li^+^, K^+^ etc.)^39,45^ or synthesized in the presence of ammonium containing precursors^35^ and hydrazine hydrate^46^. According to previous theoretical calculations, the presence of the positive counterions could cause an increase of the electron density of the *d*-orbital of the transition metals, leading to the stabilization of the 1T or distorted 1T phase^47^. Therefore, the realization of the distorted 1T-Sn_0.5_W_0.5_S_2_ in our present work might also be a result of the intercalated NH_4_^+^ cations from CS(NH_2_)_2_ and (NH_4_)_10_H_2_(W_2_O_7_)_6_ used in the synthesis solution.

### Formation process of Sn_0.5_W_0.5_S_2_/SnS_2_

**Fig. 3:**
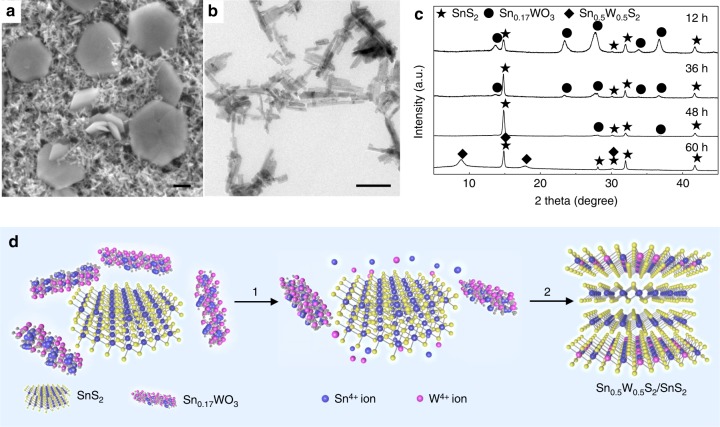
Formation process of Sn_0.5_W_0.5_S_2_/SnS_2_ heterostructures. **a** SEM image of the intermediate product obtained after the reaction proceeded for 12 h (scale bar, 200 nm). **b** TEM image of typical Sn_0.17_WO_3_ nanorods (scale bar, 100 nm). **c** XRD patterns of the intermediate products obtained at different reaction intervals. **d** Schematic illustration of the formation process of Sn_0.5_W_0.5_S_2_/SnS_2_ heterostructures. SnS_2_ nanoplates and Sn_0.17_WO_3_ nanorods formed together during the initial 12 h. After that the Sn_0.17_WO_3_ nanorods began to decompose (step 1), providing additional W and Sn ions for Sn_0.5_W_0.5_S_2_ nanosheets to grow on the surfaces of the SnS_2_ nanoplates (step 2)

To investigate the formation process of the Sn_0.5_W_0.5_S_2_/SnS_2_ vertical heterostructures, intermediate products were collected at different reaction intervals for characterizations. At the beginning of the synthesis process, the precursors, i.e., (NH_4_)_10_H_2_(W_2_O_7_)_6_ and SnCl_4_·5H_2_O, reacted to produce Sn(HWO_4_)_2_*•n*H_2_O amorphous particles upon mixing at 80 °C (Supplementary Fig. 12)^48^, which were subsequently heated up to 220 °C under hydrothermal conditions. After the reaction had proceeded for 12 h at 220 °C, nanorods with lengths of 10–100 nm were observed together with SnS_2_ nanoplates in the solution (Fig. 3a, b). These nanorods are alloyed oxide of Sn and W with a formula of Sn_0.17_WO_3_ based on XRD pattern (ICSD No. 38043 [https://icsd.fiz-karlsruhe.de], Fig. 3c)^49^, EDX analysis (Supplementary Fig. 13a, b) and HRTEM imaging (Supplementary Fig. 13c). The reason why WS_2_ was not produced at this stage is that the bond energy of Sn–S was likely to be lower than that of W–S due to the larger ionic radius of Sn^4+^ compared to that of W^4+^^50,51^. As the reaction proceeded further, the amount of the Sn_0.17_WO_3_ nanorods decreased, and nanosheets started to form on the surfaces of the SnS_2_ nanoplates (Supplementary Fig. 14). Evidently, the evolution of the XRD patterns of the intermediate products indicates a decrease of the Sn_0.17_WO_3_ amount over time (Fig. 3c), which is accompanied with an increase of the Sn and W ion concentration in the solution based on the inductively coupled plasma mass spectrometry (ICP-MS) measurements (Supplementary Table 2). Based on our control experiments, Sn_0.17_WO_3_ nanorods could form at 180 °C and decompose at temperatures above 200 °C (Supplementary Fig. 15). This suggests that the Sn_0.17_WO_3_ nanorods forming at the beginning of the hydrothermal reaction gradually decomposed at 220 °C (step 1 in Fig. 3d), providing additional Sn and W ions with a high W/Sn ratio (>30) (Supplementary Table 2). Such a high W/Sn ratio could drive the growth of alloyed Sn_0.5_W_0.5_S_2_ nanosheets on the surface of SnS_2_ even though the formation of W–S bond is less favored compared to that of the Sn–S bond (step 2 in Fig. 3d). In addition, we also tried to extend the Sn–W binary system to Sn–Mo system, but found that, under certain conditions, Sn_1–*x*_Mo_*x*_S_2_ nanosheets grew epitaxially on SnS_2_ mainly via the edge growth. This phenomenon might be due to the different synthesis energies required for basal growth or edge growth^3^, which requires our further investigation.

### Electronic properties of Sn_0.5_W_0.5_S_2_/SnS_2_

**Fig. 4:**
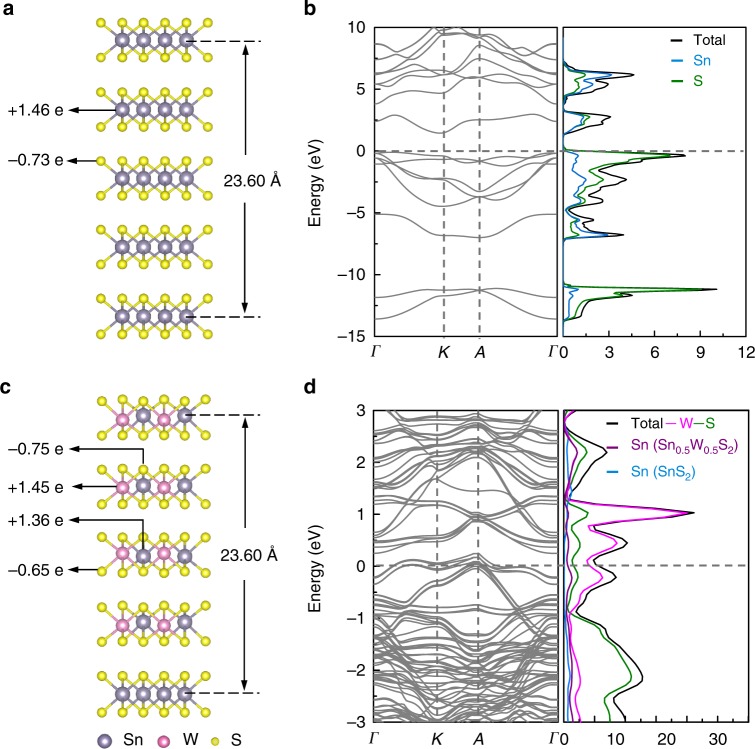
Calculated electronic structure of SnS_2_ and Sn_0.5_W_0.5_S_2_/SnS_2_ heterostructures. **a** Optimized crystal structure with calculated Bader charges. **b** Band structure and DOS of a 5-layer SnS_2_, showing an indirect band gap near the Fermi level. **c** Optimized crystal structure with calculated Bader charges. **d** Band structure and DOS of a four-layer 1T-Sn_0.5_W_0.5_S_2_ on a monolayer 1T-SnS_2_, showing the intrinsic metallic characteristic. The Fermi level is assigned at 0 eV

Alloying has been a powerful approach to tune the bandgaps of TMCs or to achieve their semiconductor-to-metal transitions^9,52^. However, alloying between layered TMC and post-TMC has not been reported so far, and thus the electronic properties of such alloys could be of great interest for potential applications. DFT calculations were performed to understand the electronic properties of the distorted 1T-Sn_0.5_W_0.5_S_2_ deposited on 1T-SnS_2_ with and without NH_4_^+^ intercalation. The detailed results of the optimized crystal structures, calculated Bader charges, band structures and density of states (DOS) are shown in Fig. 4, Supplementary Figs 16 and 17 and Supplementary Table 1. In contrast to a five-layer semiconducting 1T-SnS_2_ which shows no DOS at the Fermi level (Fig. 4b), a four-layer distorted 1T-Sn_0.5_W_0.5_S_2_ on a monolayer 1T-SnS_2_ exhibits intrinsic metallic behavior with observable DOS at the Fermi level, dominantly contributed from the W and S atoms and slightly from the Sn atoms (Fig. 4d). The calculated Bader charge of W atoms in distorted 1T-Sn_0.5_W_0.5_S_2_ is 0.20 e higher compared to that in 1T-WS_2_, whereas the Bader charge of Sn atoms in distorted 1T-Sn_0.5_W_0.5_S_2_ is 0.10 e lower compared to that in 1T-SnS_2_ (Fig. 4a, c and Supplementary Fig. 16). This suggests a charge redistribution in the Sn_0.5_W_0.5_S_2_ alloy by charge transfer from W to Sn atoms^53^. Similarly, the distorted 1T-Sn_0.5_W_0.5_S_2_ with intercalated NH_4_^+^ ions (20 mol%) also shows the metallic behavior (Supplementary Fig. 17). To experimentally verify the calculated results, the electronic properties of the SnS_2_ nanoplates and Sn_0.5_W_0.5_S_2_/SnS_2_ heterostructures were measured by fabrication of back-gated thin film field effect transistors (Supplementary Fig. 18). The drain current (*I*_d_) vs. drain-source voltage (*V*_ds_) curves at varied gate voltages reveal that the SnS_2_ nanoplates are typical n-type semiconductors (Supplementary Fig. 18a, b)^12^. In contrast, the *I*–*V* curves of films prepared from Sn_0.5_W_0.5_S_2_/SnS_2_ heterostructures are almost insensitive to gate voltages (Supplementary Fig. 18c, d), suggesting the metallic charge transport through the Sn_0.5_W_0.5_S_2_ nanosheets, consistent with the theoretical predictions.

**Fig. 5:**
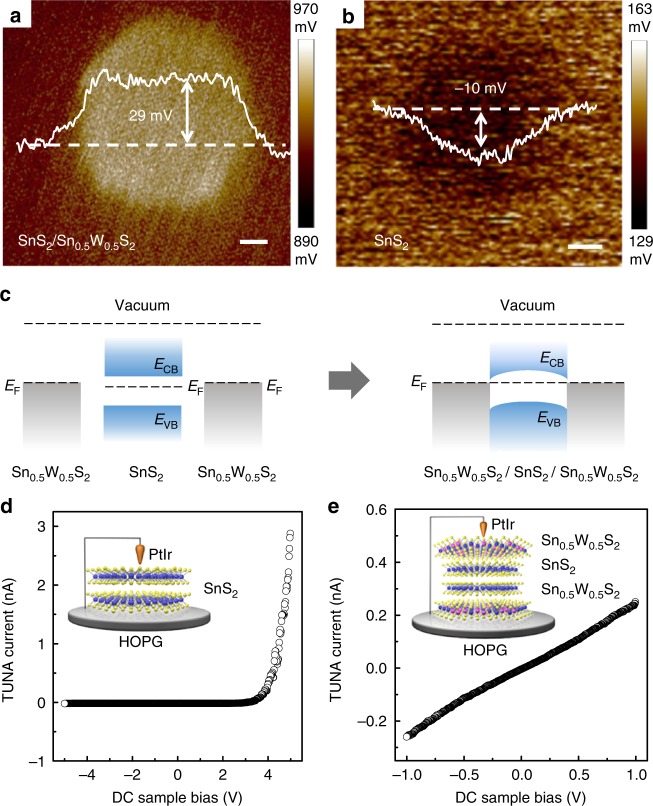
KPFM and TUNA analyses of SnS_2_ and Sn_0.5_W_0.5_S_2_/SnS_2_ heterostructures. 2D potential images of **a** a typical Sn_0.5_W_0.5_S_2_/SnS_2_ heterostructure (scale bar, 200 nm) and **b** a typical SnS_2_ nanoplate deposited on a SiO_2_/Si substrate coated with a thin film of Au/Cr (i.e., Au/Cr/SiO_2_/Si) (scale bar, 100 nm). **c** Schematic band alignment diagram for Sn_0.5_W_0.5_S_2_ and SnS_2_ before and after contact. *E*_F_, *E*_CB_, and *E*_VB_ denote Fermi level, conduction band and valence band, respectively. *I*–*V* curves measured with TUNA for **d** a SnS_2_ nanoplate and **e** a Sn_0.5_W_0.5_S_2_/SnS_2_ heterostructure, under a constant force and an applied bias voltage that was linearly ramped down

To exmamine the interface property between a SnS_2_ nanoplate and the surface deposited Sn_0.5_W_0.5_S_2_, its surface potential, which corelates to its work function, was analyzed by KPFM in air^21^. A PtIr tip was used as the probe, and a SiO_2_/Si substrate sputtered with Au/Cr with a theoretical work function (*ɸ*_Au_) of 5.100 eV was used as the potential reference. The 2D potential image of a typical Sn_0.5_W_0.5_S_2_/SnS_2_ heterostructure is shown in Fig. 5a, where the color variation reflects the local surface potential difference (Δ*V* = *ɸ*_Au_ − *ɸ*_sample_) (details on the calculation of the surface potentials are given in the Methods section). It can be seen that the surface potential of the Sn_0.5_W_0.5_S_2_/SnS_2_ heterostructure is 0.029 V lower than that of Au to give an estimated work function of 5.071 eV (Fig. 5a). On the other hand, an SnS_2_ nanoplate exhibits a work function of 5.110 eV (Fig. 5b). The decrease in surface potential after deposition of Sn_0.5_W_0.5_S_2_ on SnS_2_ suggests that the metallic Sn_0.5_W_0.5_S_2_ possesses a lower work function than that of the n-type SnS_2_. Although based on DFT calcuations, the work function of distorted 1T-Sn_0.5_W_0.5_S_2_ is 5.62 eV which is much higher than that of SnS_2_, it can be substantially lowered to 2.87 eV by introducing an NH_4_^+^ intercalation with a molar concentration of about 20% (Supplementary Table 1). Such work function modulation induced by doping or chemical absorbates has also been reported previously^54^. This also explains why the experimentally measured work function of Sn_0.5_W_0.5_S_2_ nanosheets with partial NH_4_^+^ intercalation was lower than that of SnS_2_. Therefore, an ohmic contact should form at the Sn_0.5_W_0.5_S_2_/SnS_2_ heterointerface (Fig. 5c), affording a low charge transfer resistance. This was further confirmed by measuring *I*–*V* curves on individual SnS_2_ nanoplates or Sn_0.5_W_0.5_S_2_/SnS_2_ heterostructures deposited on highly oriented pyrolytic graphite (HOPG) with TUNA (Fig. 5d, e). The *I*–*V* curve for a SnS_2_ nanoplate is highly asymmetric and the onset of the current rectification is at 3.50 V (Fig. 5d), indicating the presence of Schottky barrier at the PtIr tip/n-type SnS_2_ interface, provided that the work function for PtIr, HOPG, and SnS_2_ is 5.50, 4.60, and 5.11 eV, respectively^55,56^. In sharp contrast, the *I*–*V* curve for Sn_0.5_W_0.5_S_2_/SnS_2_ is almost linear and symmetric with respect to 0 V, suggesting that the contact at SnS_2_/Sn_0.5_W_0.5_S_2_ interface is ohmic-like (Fig. 5e)^57^.

### Sn_0.5_W_0.5_S_2_/SnS_2_ for photodetectors

**Fig. 6:**
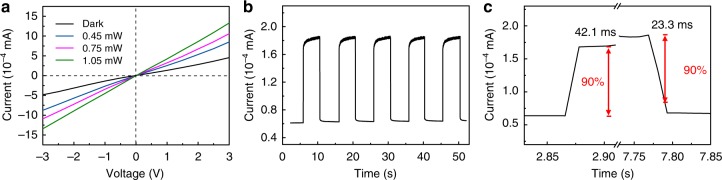
Photodetector performance of Sn_0.5_W_0.5_S_2_/SnS_2_ heterostructures. **a** *I*–*V* curves at different light intensity, **b** temporal photocurrent response and **c** a zoom-in view of the temporal photocurrent response of a photodetector based on Sn_0.5_W_0.5_S_2_/SnS_2_ heterostructures. The light source used for all measurements was a 405 nm laser

The advantage of the facile charge transport across the ohmic-like heterointerfaces was demonstrated in fabrication of thin film photodetectors based on the Sn_0.5_W_0.5_S_2_/SnS_2_ heterostructures. Figure 6a shows the *I*–*V* curves of the device under 405 nm laser illumination with power intensity varied from 0.45 to 1.05 mW. A clear rise of the photocurrent with increasing illumination intensity was observed, indicating the effective conversion of photon flux to photogenerated carriers. In addition, the Sn_0.5_W_0.5_S_2_/SnS_2_ photodetector showed symmetric and linear *I*–*V* plots, which is in sharp contrast to the non-linear *I*–*V* curves observed for SnS_2_-based device (Supplementary Fig. 19a). This further indicates the low resistance contact formed between the semiconducting and metallic components in Sn_0.5_W_0.5_S_2_/SnS_2_. The temporal photoresponse of the photodetectors was measured as well as shown in Fig. 6b, c and Supplementary Figs. 19b, c. The Sn_0.5_W_0.5_S_2_/SnS_2_ photodetector showed an abrupt rise of photocurrent with a fast response time of 42.1 ms (defined as the time required to increase 90% from the minimum to maximum current density), which is comparable and outperforms some previously reported TMC based photodetectors^58,59^. This value is also about 50 times shorter than that of the SnS_2_-based photodetector (2.10 s) (Supplementary Fig. 19c). Such markedly shortened response time suggests the rapid transport of charge carriers across the Sn_0.5_W_0.5_S_2_/SnS_2_ heterointerfaces^60,61^. Note that a relatively large dark current and thus a much reduced on/off ratio were observed for the Sn_0.5_W_0.5_S_2_/SnS_2_-based device as compared with the SnS_2_ device. This was due to the metallic nature of the Sn_0.5_W_0.5_S_2_ nanosheets. The similar phenomenon was reported previously in photodetectors based on graphene composites^62,63^.

### Sn_0.5_W_0.5_S_2_/SnS_2_ for gas sensing

**Fig. 7:**
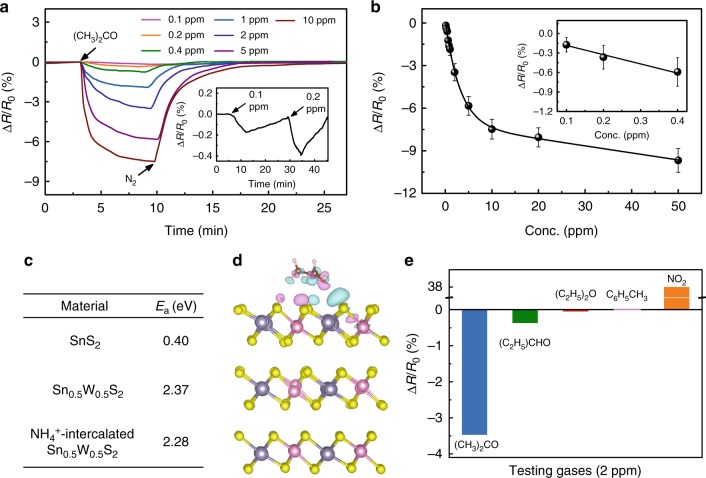
Gas sensing performance of Sn_0.5_W_0.5_S_2_/SnS_2_ heterostructures. **a** Response–recovery curves of a typical chemiresistive sensor fabricated from Sn_0.5_W_0.5_S_2_/SnS_2_ heterostructures in response to acetone gas with increasing concentrations. Inset: zoom-in response of the sensor towards 0.1 and 0.2 ppm acetone. **b** Normalized change of resistance of Sn_0.5_W_0.5_S_2_/SnS_2_ sensors at various acetone concentrations. Inset: zoom-in normalized change of resistance at low acetone concentrations. Each error bar indicates the standard deviation of the change of resistance for 5 experimental replicates. **c** Calculated adsorption energy, *E*_a_ (eV), of acetone on different sensing materials. **d** Side view of the fully relaxed structural model of Sn_0.5_W_0.5_S_2_ with surface adsorption of an acetone molecule. Cyan regions indicate charge accumulation, while pink regions represent charge depletion. **e** Comparison of the responses of the sensor towards different gases, including acetone, diethyl ether, propanal, toluene, and NO_2_

The presence of a metallic component in a thin film channel may pose some limitation to its optoelectronic performance, such as the relatively large dark current observed in the aforementioned Sn_0.5_W_0.5_S_2_/SnS_2_ photodetector. However, the metallic structure might become an advantage for applications like sensors. As another proof of concept demonstration of the advantage of solution-processible functional materials, the Sn_0.5_W_0.5_S_2_/SnS_2_ heterostructures were deposited on Au interdigitated electrodes to fabricate chemiresistive gas sensors for detection of volatile organic compounds such as acetone, which is a potential biomarker for diabetes and lung cancer^64^. For comparison, SnS_2_-based gas sensors were also fabricated. The response–recovery curves of the gas sensors were measured under gas flows with increasing concentrations (typically 0.1–50 ppm) at room temperature (Fig. 7a, b and Supplementary Figs. 20 and 21). Taking sensing of acetone for example, the resistance of the Sn_0.5_W_0.5_S_2_/SnS_2_ sensor decreased upon exposure to acetone and the decrease in resistance (Δ*R* = *R*_a_ – *R*_0_) with increasing acetone concentration (Fig. 7a, b and Supplementary Fig. 20). A minimum detectable concentration of 0.1 ppm was achieved. This is 20 times lower compared to that of the sensor based on SnS_2_ which only afforded a minimum detectable concentration of 2 ppm (Supplementary Fig. 21), and to the best of our knowledge, outperforms other reported metal sulfide/oxide chemiresistive acetone sensors. More importantly, our sensor showed the best sensitivity (i.e., sensing response, Δ*R*/*R*_0_) at 100 ppb levels among all reported chemiresistive sensors operating at room temperature (Supplementary Table 3). Furthermore, as shown in Supplementary Fig. 22, a typical Sn_0.5_W_0.5_S_2_/SnS_2_ sensor was repeatedly exposed to 1 ppm acetone and then back to N_2_ gas for 10 cycles and showed an almost constant sensing response of 1.88 ± 0.07% (by taking standard deviation of the results from 10 cycles), indicating its good repeatability.

The improved sensitivity after deposition of Sn_0.5_W_0.5_S_2_ nanosheets on SnS_2_ nanoplates could be attributed to the following reasons. First, the formation of an ohmic-like contact between Sn_0.5_W_0.5_S_2_ and SnS_2_ that allowed rapid charge transfer across the metal–semiconductor interface partly led to a 35 times reduction in background noise and thus a much higher signal to noise (S/N) ratio (Supplementary Fig. 23)^27^. Second, it can be noted that, the response/recovery time of the Sn_0.5_W_0.5_S_2_/SnS_2_ gas sensor was longer than that of the SnS_2_ gas sensor (Fig. 7a and Supplementary Fig. 21), pointing to a chemical adsorption-related sensing pathway^64,65^. To confirm this, we calculated the adsorption energy of acetone on the different sensing materials (Fig. 7c). The adsorption energy of acetone on Sn_0.5_W_0.5_S_2_ is 2.37 eV is much larger compared to that on SnS_2_ (0.40 eV). This indicates that acetone molecules interact more strongly with Sn_0.5_W_0.5_S_2_. It is worth noting that the NH_4_^+^-intercalation did not significantly change the adsorption ability of Sn_0.5_W_0.5_S_2_ nanosheets towards acetone, with the adsorption energy slightly reduced by 0.09 eV. As further shown in Fig. 7d, there is an obvious charge accumulation on Sn_0.5_W_0.5_S_2_ due to charge transfer from the absorbed acetone molecule. Third, one of the advantages of using low-dimensional materials in gas sensing as compared with bulk crystals is their large specific surface areas, which are beneficial for providing large interfaces for channel–gas interaction^25^. Indeed, the deposition of wrinkled Sn_0.5_W_0.5_S_2_ nanosheets on SnS_2_ nanoplates increased the specific surface areas from typically 6.25 to 11.37 m²/g based on the Brunauer–Emmett–Teller (BET) measurements as shown in Supplementary Fig. 24. However, in spite of the beneficial effects from the large specific surface area of the wrinkled Sn_0.5_W_0.5_S_2_ nanosheets and their strong interaction with acetone, increasing the amount of Sn_0.5_W_0.5_S_2_ nanosheets on SnS_2_ did not further improve the sensing performance, but on the contrary, led to poorer sensitivity with a minimal detectable concentration of only 1 ppm (Supplementary Fig. 25). This suggests that the concentration of the metallic phase present in the hybrid sensing film should not be too high, otherwise, the gas-induced doping effect on the semiconducting SnS_2_ would be substantially weakened. Therefore, the presence of the semiconductor/metal heterostructures with a low charge transfer barrier, combined with sufficient active surfaces for strong gas adsorption are important in achieving low sensitivity in our thin film based gas sensors.

The selectivity of the Sn_0.5_W_0.5_S_2_/SnS_2_ sensor was also investigated by comparing its sensing response towards acetone with other electron donating gases like diethyl ether and propanal, neutral gas like toluene, and electron withdrawing gas like NO_2_ (Fig. 7e, Supplementary Fig. 26). The response of the Sn_0.5_W_0.5_S_2_/SnS_2_ sensor towards diethyl ether and propanal was 10 times lower compared to that towards acetone at 2 ppm. This is largely due to the weaker electron donating ability of diethyl ether and propanal compared to that of acetone (Fig. 7e). Under the exposure of toluene, the sensor showed no response. In sharp contrast, when responding to NO_2_, an electron withdrawing gas, the sensor showed increased resistance (Fig. 7e, Supplementary Fig. 26e), agreeing with the fact that SnS_2_ is an n-type semiconductor (Supplementary Fig. 18b).

## Discussion

The epitaxial growth of metallic Sn_0.5_W_0.5_S_2_ nanosheets on the surfaces of SnS_2_ nanoplates were realized via a solution-phase epitaxial deposition process. Importantly, an alloyed metal oxide (i.e., Sn_0.17_WO_3_) was identified as an intermediate product which formed at 180 °C and decomposed subsequently at 220 °C to provide additional Sn and W ions with a high W/Sn ratio of about 30. We proposed that this high W/Sn concentration could drive the formation of alloyed Sn_0.5_W_0.5_S_2_ nanosheets despite the fact that the formation of Sn–S bond requires less energy compared to that of the W–S bond. Although 1T-SnS_2_ is a semiconductor, alloyed Sn_0.5_W_0.5_S_2_ showed 83% distorted 1T structure, which is metal-like as predicted by theoretical calculations. KPFM and TUNA measurements on a Sn_0.5_W_0.5_S_2_/SnS_2_ heterostructure suggested the formation of ohmic-like contact at the heterointerface, resulting in a low charge transfer resistance. The rapid charge transport at the Sn_0.5_W_0.5_S_2_/SnS_2_ heterointerface allowed for the fabrication of fast photodetector with a short response time of 42 ms. Additionally, when the Sn_0.5_W_0.5_S_2_/SnS_2_ heterostructures were fabricated into thin films for gas sensing, a much enhanced signal-to-noise ratio was achieved partly due to the presence of metallic Sn_0.5_W_0.5_S_2_ layers. Furthermore, Sn_0.5_W_0.5_S_2_ showed much enhanced surface adsorption of acetone than SnS_2_ based on theoretical calculations. As a consequence, selective and sensitive detection of acetone was achieved with an ultralow minimum detectable concentration of 100 ppb at room temperature. This, combined with large sensing responses at 100 ppb levels outperforms previously reported room-temperature chemiresistive sensors. Our use of semiconducting SnS_2_ nanoplates as synthesis templates for the solution-phase epitaxial growth of metallic Sn_0.5_W_0.5_S_2_ nanosheets demonstrates a promising way towards the facile, economic and high-yield preparation of functional hybrid nanomaterials. Synthesis of layered materials through alloying among elements with distinct electronic and chemical properties is expected to bring about more materials and unusual phenomena.

## Methods

### Materials

Ammonium tungstate hydrate ((NH_4_)_10_H_2_(W_2_O_7_)_6_, 99.99%), ammonium molybdate tetrahydrate ((NH_4_)_6_Mo_7_O_24_·4H_2_O, 99.0%), thiourea (CS(NH_2_)_2_, 99.0%) and tin tetrachloride hydrate (SnCl_4_·5H_**2**_O, 99.9%) were purchased from Sigma-Aldrich (Shanghai, China). Ethanol (C_2_H_5_OH, ACS, 99.9%) was purchased from J&K chemical (Shanghai, China). The gaseous analytes (CH_3_COCH_3_, (CH_3_CH_2_)_2_O, CH_3_CH_2_CHO, C_6_H_5_CH_3_, and NO_2_) which were diluted with N_2_ gas at concentrations of 1000 ppm were purchased from Nanjing Teqi Co., Ltd. All chemicals were used as received without further purification. The deionized (DI) water was purified using Milli-Q3 System (Millipore, France).

### Preparation of SnS_2_ nanoplates

In a typical process, 0.25 mmol of SnCl_4_·5H_2_O and 3.75 mmol of CS(NH_2_)_2_ were dissolved in 19.45 mL DI water and stirred for 2 h to form a homogeneous solution. This solution was transferred to a 25 mL Teflon-lined stainless steel autoclave, heated to 220 °C in an electrical oven and then maintained at this temperature for 12 h before being cooled down naturally to room temperature. The obtained product was centrifuged at 8000 rpm for 10 min, and the precipitate was washed with DI water for three times before further characterization.

### Preparation of Sn_0.5_W_0.5_S_2_/SnS_2_ heterostructures

In a typical process, 0.25 mmol of (NH_4_)_10_H_2_(W_2_O_7_)_6_, 7.5 mmol of CS(NH_2_)_2_, and 0.5–0.625 mmol of SnCl_4_·5H_2_O were dissolved in 19.45 mL DI water and stirred at 80 °C for 2 h to form a homogeneous solution. This solution was then transferred to a 25 mL Teflon-lined stainless steel autoclave, heated to 220 °C in an electrical oven and then maintained at this temperature for 60 h before being cooled down naturally to room temperature. The obtained product was centrifuged at 8000 rpm for 10 min, and the precipitate was washed with DI water for three times before further characterization.

### Characterizations

Scanning electron microscope (SEM, JEOL JSM-7800F, Japan), transmission electron microscope (TEM, JEOL 2100Plus, Japan) and high resolution transmission electron microscope (HRTEM, JEOL 2100 F, Japan) coupled with energy dispersive X-ray (EDX) spectroscope were used to investigate the compositional, morphological and structural features of the samples. X-ray diffraction (XRD, Rigaku SmartLab, Japan) was performed using CuKα radiation (λ = 1.54 Å). X-ray photoelectron spectroscopy (XPS, PHI 5000 VersaProbe, Japan) measurements were conducted on the different metal sulfide nanostructures, and the binding energies were corrected for specimen charging effects using the C 1 s level at 284.6 eV as the reference. Raman spectra (Horiba HR800, France) of the samples were collected with a 532 nm laser. Semiconductor parameter analyzer (Tektronix Keithley 4200, America) and probe station (Lake Shore TTPX, America) were used to investigate the semiconductor properties of the samples. A commercial atomic force microscope (AFM, Dimension ICON with Nanoscope V controller, Bruker) was used to investigate the electrical properties of the individual nanostructures in air. Inductively coupled plasma mass spectrometry (ICP-MS, Agilent 7700×, America) was used to measure the concentration of Sn and W ions in the synthesis solution. Brunauer–Emmett–Teller (BET, Micromeritics, ASAP2460, USA) measurements were carried out to determine the specific surface area and pore size distribution of various samples.

### Semiconducting property characterization

After Au (50 nm)/Cr (30 nm) drain and source electrodes were deposited onto a SiO_2_ (285 nm)/Si substrate via thermal evaporation through a shadow mask, SnS_2_ nanoplates or Sn_0.5_W_0.5_S_2_/SnS_2_ heterostructures in water were drop-casted onto the substrate, acting as the channel to connect the drain and source electrodes with a channel length of 11 μm. The semiconducting properties of the channel materials were then characterized using a Keithley 4200 semiconductor characterization system operating at 77 K in vacuum (5 × 10^−5^ Torr).

### KPFM and TUNA measurements

After Au (50 nm)/Cr (30 nm) was coated onto a SiO_2_ (285 nm)/Si substrate via thermal evaporation, SnS_2_ nanoplates (or Sn_0.5_W_0.5_S_2_/SnS_2_ heterostructures) in water were drop-casted onto the substrate. A KPFM (Dimension ICON with Nanoscope V controller, Bruker) was then used to characterize the surface potential of the SnS_2_ nanoplates (or Sn_0.5_W_0.5_S_2_/SnS_2_ heterostructures) at ambient conditions. The contact potential difference between the tip (PtIr) and the sample surface (*V*_CPD_), which is also referred to as the surface potential can be calculated by using the following equations:

1 $$V_{{\mathrm{CPD}}}{\mathrm{ = }}\frac{1}{e}\left( {\varphi _{\mathrm{t}} - \varphi _{\mathrm{f}}} \right)$$

$$\Delta V_{{\mathrm{CPD}}} = \Delta V_{{\mathrm{CPD}}}\left( {{\mathrm{film}}} \right) - \Delta V_{{\mathrm{CPD}}}\left( {{\mathrm{substrate}}} \right)$$

2 $$\Delta V_{{\mathrm{CPD}}}\,=\,\frac{1}{e}\left( {\varphi _{\mathrm{t}} - \varphi _{\mathrm{f}}} \right) - \frac{1}{e}\left( {\varphi _{\mathrm{t}} - \varphi _{\mathrm{s}}} \right) = \frac{1}{e}\left( {\varphi _{\mathrm{s}} - \varphi _{\mathrm{f}}} \right)$$

where *φ*_t_, *φ*_s_, and *φ*_f_ represent the work functions of the probe tip, the substrate, and the sample film, respectively.

PeakForce Tunneling atomic force microscopy (TUNA, Dimension ICON with Nanoscope V controller, Bruker) was used to investigate the current–voltage (*I*–*V*) characteristics of individual SnS_2_ nanoplates or Sn_0.5_W_0.5_S_2_/SnS_2_ heterostructures. During the measurement, the PtIr tip was pressed against the sample with a constant force, feedback was switched to contact mode, and the voltage was linearly ramped up and down while the current signal was collected. Analysis of the *I*–*V* curves was performed with the Nanoscope Analysis software.

### Fabrication of photodetector and photoresponse measurements

Au (15 nm in thickness) interdigitated electrodes with a 10 nm spacing were deposited onto a SiO_2_ (300 nm)/Si substrate via magnetron sputtering through a shadow mask. After that, 0.5 µL of a concentrated dispersion (6.0 mg/mL) of the Sn_0.5_W_0.5_S_2_/SnS_2_ heterostructures or SnS_2_ nanoplates was drop-casted on the electrodes.

The current–voltage (*I*–*V*) and the current–time (*I*–*t*) curves of the photodetectors were measured on a semiconductor characterization system (Keithley 4200, USA) in air at room temperature. A 405 nm laser was used for all the measurements. The actual power intensity was measured by a power meter (LP1, Sanwa Electric Instrument Co., Ltd., Japan).

### Fabrication of chemiresistive sensors and gas sensing tests

Chemiresistive gas sensors were fabricated based on SnS_2_ nanoplates or Sn_0.5_W_0.5_S_2_/SnS_2_ heterostructures for sensing of various gases, including CH_3_COCH_3_, (CH_3_CH_2_)_2_O, CH_3_CH_2_CHO, C_6_H_5_CH_3_, and NO_2_. Typically, a drop of 100 μL aqueous solution containing 10 mM as-prepared SnS_2_ or Sn_0.5_W_0.5_S_2_/SnS_2_ was drop-casted onto an Au interdigitated electrode (with 0.1 mm spacing over a 2 × 1 cm^2^ area, Changchun Mega Borui Technology Co., Ltd) and then dried in oven at 60 °C. The gas sensing test was performed in an air-tight chamber with electrical feedthroughs at room temperature (25 °C). A constant current was applied to the sensor electrode, and the variation of the sensor resistance was monitored and recorded with the changes in the gas environment using a data acquisition system (34972A, Agilent) with a 20 channel multiplexer (34901A, Agilent). A typical sensing measurement cycle consisted of three sequential steps: (1) a dried N_2_ flow was introduced into the chamber to record a baseline resistance (*R*_0_); (2) a target gas, e.g., acetone, balanced in N_2_ was introduced, and the concentration increased (0.1–50 ppm) with progressive cycles; and (3) when the resistance of the sensor reached equilibrium in the target gas (*R*_a_), the target gas was replaced by N_2_ to allow the resistance of the sensor to return to *R*_0_. All gas flows were set at 500 sccm, precisely controlled by using mass-flow controllers.

### Computational methods

All the computations were performed with Vienna Ab initio simulation package (VASP) which is based on the density functional theory (DFT)^66,67^. The exchange-correlation interaction uses the general gradient approximation (GGA) formulated by Perdew–Burke–Ernzerhof (PBE)^66^. All electron interactions were described with projector augmented wave (PAW) pseudo potentials. Long-range dispersion corrections have been considered within the DFT-D2 method. The dispersion coefficient C_6_ and van der Waals radius *R*_0_ for H, C, N, O, S, Sn and W used in our DFT-D2 method were taken from previous reports^68,69^. The scale factor S_6_ was set to 0.75 because the GGA-PBE function was employed. An 11 × 11 × 1 k-point mesh was used for the interaction of the Brillouin-zone. The cutoff energy for the plane wave basis set was restricted to 400 eV, and a vacuum region of at least 12 Å was used in building the slab models. The convergence threshold was set as 10^−4^ eV in energy and 0.02 eV/Å in force, respectively. As shown in Fig. 4c, a four-layer 1T-Sn_0.5_W_0.5_S_2_ on a monolayer 1T-SnS_2_ was constructed with a 2 × 2 supercell, which contains 24 Sn, 16 W and 80 S atoms, respectively. Three NH_4_^+^ ions were introduced in the four-layer 1T-Sn_0.5_W_0.5_S_2_ on a monolayer 1T-SnS_2_ system (Supplementary Fig. 10). All atoms in the structure were fully relaxed to optimize without any restriction, and the convergence threshold was set as 10^−4^ eV in energy and 0.02 eV/Å in force, respectively. The optimized lattice constants and work functions (*Φ*) were summarized in Supplementary Table 1. The experimental lattice constant (*a* = 3.46 Å) was used in DFT calculations for the in-plane periodicity of the four-layer 1T-Sn_0.5_W_0.5_S_2_ on a monolayer 1T-SnS_2_ without and with intercalated NH_4_^+^ ions. To evaluate the stability of the adsorption of an acetone molecule on a three-layer 1T-SnS_2_ and three-layer 1T-Sn_0.5_W_0.5_S_2_ without and with intercalated NH_4_^+^ ions (each system contains a 4 × 4 supercell), the adsorption was defined by △*E*_a_ _=_ *E*_acetone_ + *E*_substrate_ − *E*_acetone–substrate_ (where *E*_acetone–substrate_ is the total energy of the acetone/substrate compound systems, whereas *E*_acetone_ and *E*_substrate_ are the energy of the isolated acetone molecule, and the total energies of upper two-layer relaxed and bottom-layer fixed 1T-SnS_2_ or distorted 1T-Sn_0.5_W_0.5_S_2_ without or with intercalated NH_4_^+^ ions systems, respectively. VESTA was used for preparation of the structure models^70^.

## Electronic supplementary material

Supplementary Information

Peer Review File

## Data Availability

The data that support the findings of this study are available from the corresponding author on request.
