# Supplementary material for: Realization of vertical metal semiconductor heterostructures via solution phase epitaxy
Source: Nat Commun. 2018 Sep 6;9:3611. doi: 10.1038/s41467-018-06053-z (PMC6127337; doi:10.1038/s41467-018-06053-z)
Supplement: Supplementary file 1 — Supplementary Information [file 41467_2018_6053_MOESM1_ESM.pdf]

Realization of vertical metal/semiconductor heterostructures via solution-phase epitaxy

Wang *et al.*

Supplementary Figures

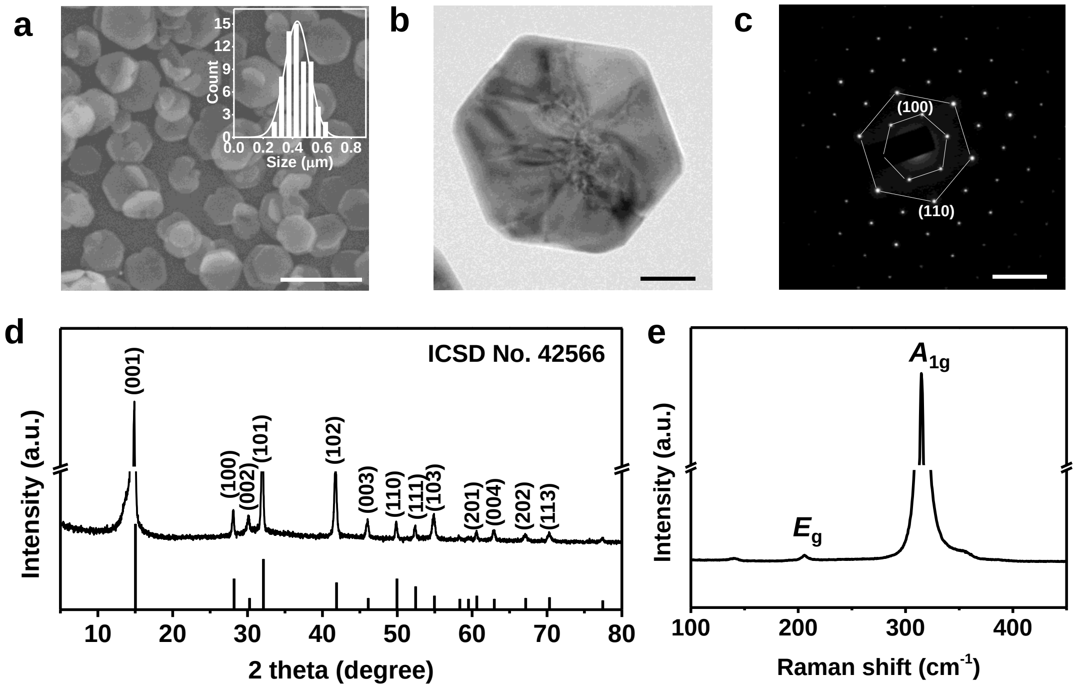

Supplementary Figure 1. (a) SEM image (scale bar, 500 nm), (b) TEM image (scale bar, 100 nm), (c) SAED pattern (scale bar, 5 nm^{-1}), (d) XRD pattern and (e) Raman spectrum of SnS_2 nanoplates. Inset of (a): lateral size distribution of the nanoplates. Raman spectrum in (e) shows two dominant peaks at 205 and 314 cm^{-1} corresponding to the E_g and A_{1g} vibrational modes of SnS_2, respectively[1].

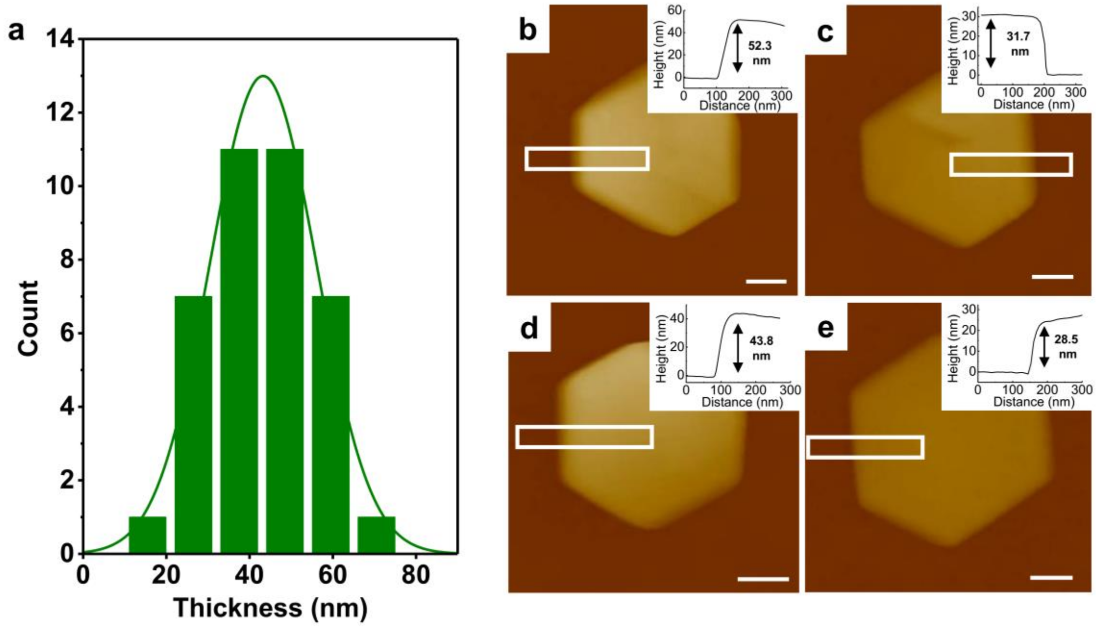

Supplementary Figure 2. (a) Thickness distribution of SnS_2 nanoplates showing a mean thickness of ~43 nm. (b-e) Examples of AFM images and height analyses of individual SnS_2 nanoplates (scale bars, 100 nm). Insets: height analysis of the region highlighted in the corresponding white rectangle.

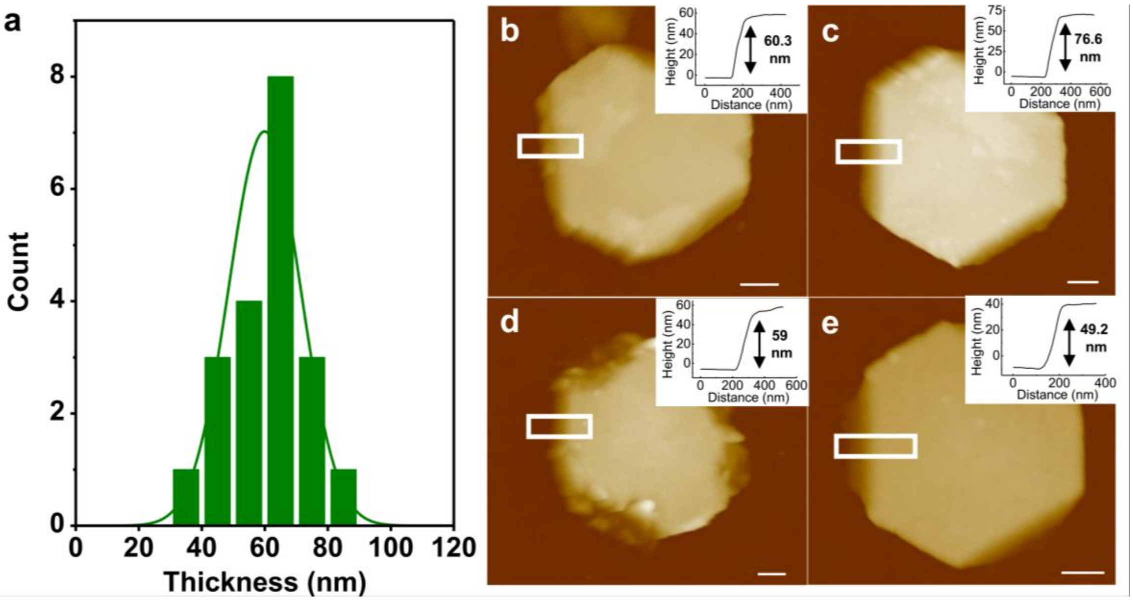

Supplementary Figure 3. (a) Thickness distribution of $Sn_{1-x}W_xS_2/SnS_2$ heterstructures, showing a mean thickness of ~60 nm. (b-e) Examples of AFM images and height analyses of individual $Sn_{1-x}W_xS_2/SnS_2$ heterstructures (scale bars, 100 nm). Insets: height analysis of the region highlighted in the corresponding white rectangle.

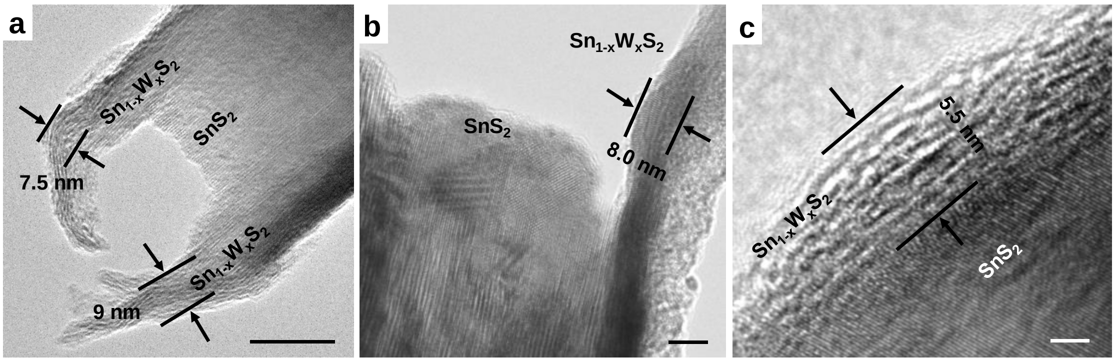

Supplementary Figure 4. (a-c) Side-view TEM images of typical $Sn_{1-x}W_xS_2/SnS_2$ heterostructures, revealing that the thickness of $Sn_{1-x}W_xS_2$ nanosheets grown on SnS_2 nanoplates is 6-9 nm. The scale bars in (a), (b) and (c) are 20 nm, 5 nm and 2 nm, respectively.

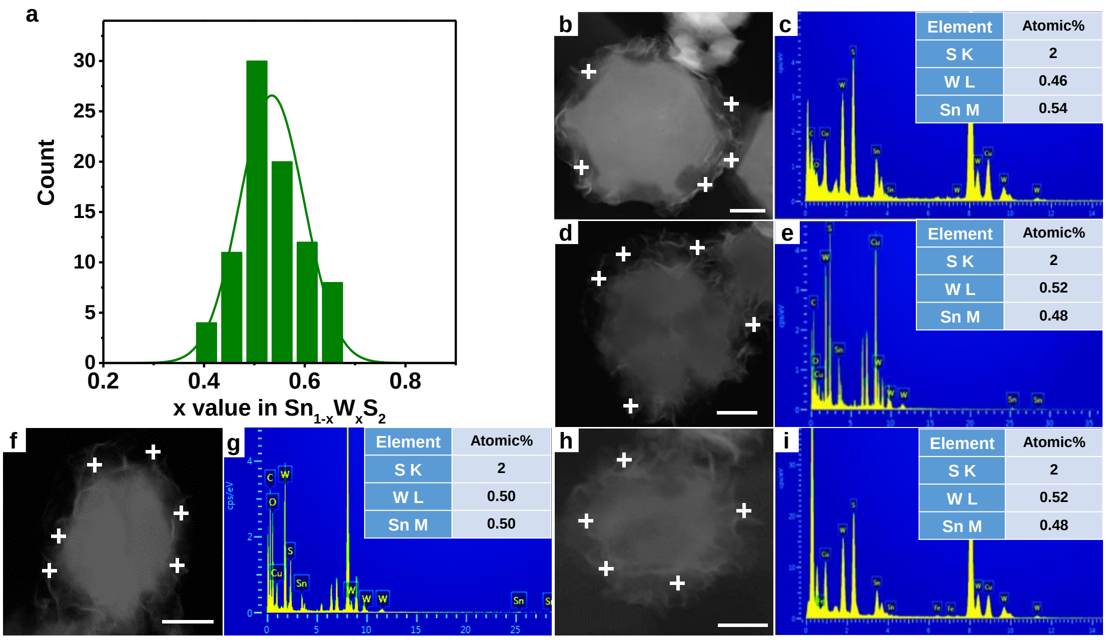

Supplementary Figure 5. (a) Distribution of x values in $Sn_{1-x}W_xS_2$ based on EDX spot analyses on the edges of several $Sn_{1-x}W_xS_2$. (b-i) Four examples of STEM images and EDX spot analyses on the edges of $Sn_{1-x}W_xS_2$ nanosheets, where the positions of the analyzed spots are highlighted in white crosses (scale bars, 200 nm). A mean value of ~0.5 was determined for x.

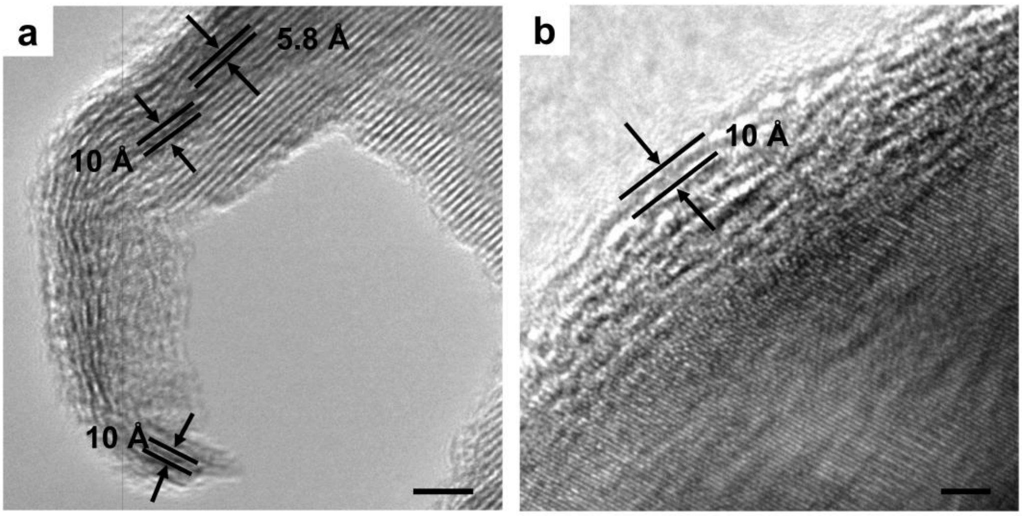

Supplementary Figure 6. (a,b) Side-view HRTEM images of typical $Sn_{0.5}W_{0.5}S_2/SnS_2$ heterostructures, revealing surface deposited $Sn_{0.5}W_{0.5}S_2$ nanosheets with varied interlayer spacings. The scale bars in (a) and (b) are 5 nm and 2 nm, respectively.

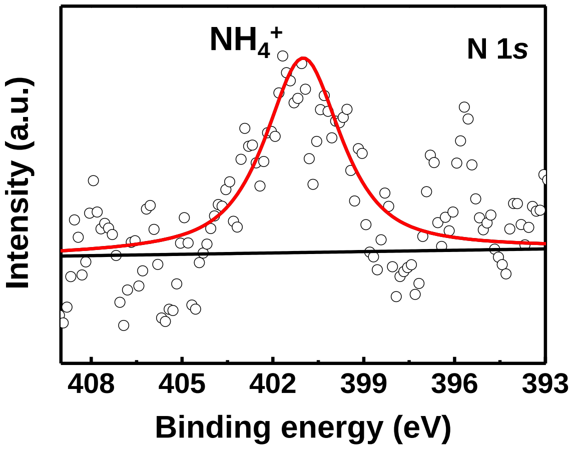

Supplementary Figure 7. XPS N 1*s* spectrum of as-prepared $Sn_{0.5}W_{0.5}S_2/SnS_2$ heterostructures, showing a peak at 401.1 eV, which can be assigned to NH_4^+ (ref. 2).

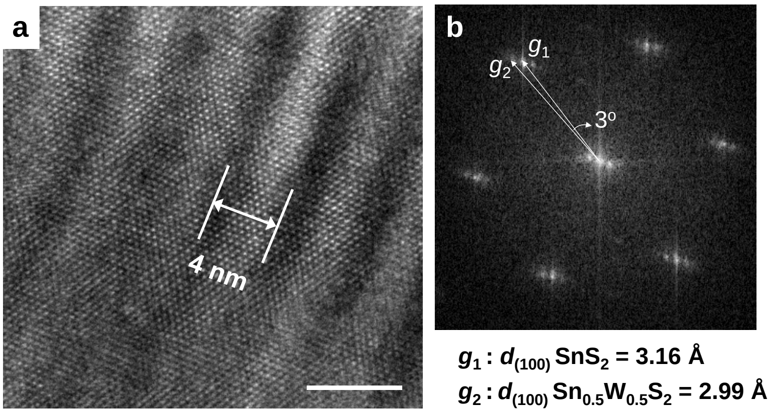

Supplementary Figure 8. (a) HRTEM image of a typical $Sn_{0.5}W_{0.5}S_2/SnS_2$ heterostructure lying flatly on a copper grid, showing a Moiré pattern with a periodicity of ~4.0 nm (scale bar, 5 nm). (b) Fast Fourier transform (FFT) diffraction pattern of (a), showing reciprocal vectors of g_1 and g_2 corresponding to the (100) planes of SnS_2 and $Sn_{0.5}W_{0.5}S_2$, with real space spacing d_1 and d_2 equaling to 3.16 and 2.99 Å, respectively. The Moiré pattern based on equation $d = \frac{d_1 d_2}{\sqrt{d_1^2 + d_2^2 - 2 d_1 d_2 \cos\theta}}$ has a calculated pattern periodicity of ~ 4.0 nm, consistent with the measurement in (a).

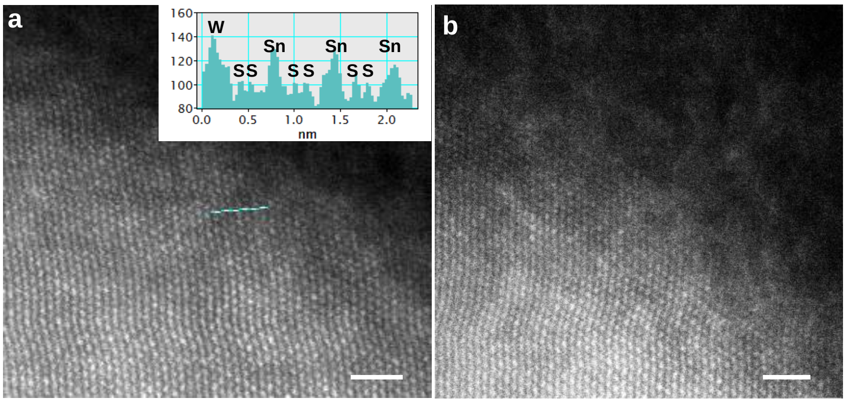

Supplementary Figure 9. (a,b) STEM images of the edge area of a typical $Sn_{0.5}W_{0.5}S_2/SnS_2$ heterostructure lying flatly on a copper grid (scale bars, 2 nm). The inset of (a) shows the contrast profile of the line marked in the image, indicating a typical metal-sulfur-sulfur-metal atomic pattern for 1T-phase-like structure[3]. From the thin area near the edge, contrast difference due to the presence of Sn and W elements can be observed.

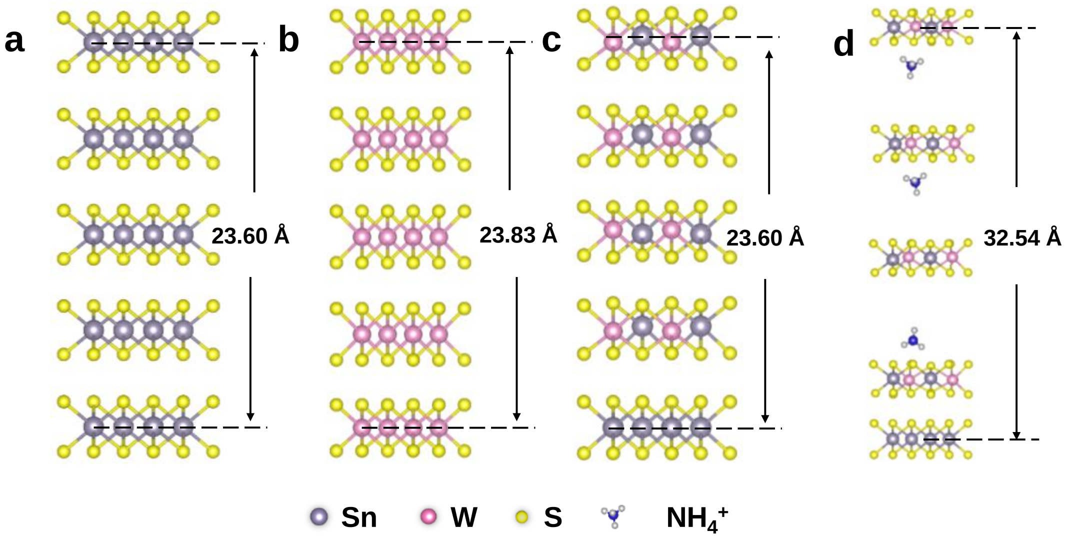

Supplementary Figure 10. Calculated structural models of (a) SnS_2, (b) 1T-WS_2, (c) a four-layer distorted 1T-$Sn_{0.5}W_{0.5}S_2$ on a monolayer 1T-SnS_2 and (d) a four-layer distorted 1T-$Sn_{0.5}W_{0.5}S_2$ on a monolayer 1T-SnS_2 with intercalated NH_4^+ ions. The optimized crystal structures of a five-layer SnS_2, a five-layer 1T-WS_2 and a four-layer distorted 1T-$Sn_{0.5}W_{0.5}S_2$ on a monolayer 1T-SnS_2 with and without NH_4^+ intercalated ions are shown in Fig. 10 and Table 1. In a 5-layer SnS_2, the Sn-S bond length is about 2.58 Å, while the W-S bond length in a 5-layer WS_2 is about 2.42 Å. The interlayer distance along the c-axis of SnS_2 is 5.90 ± 0.03 Å, shorter than that of WS_2 by ~0.10 Å. For $Sn_{0.5}W_{0.5}S_2$ without NH_4^+ intercalation, the bond lengths of Sn-S and W-S are 2.52 Å and 2.43 Å, respectively, and its interlayer distance along the c-axis is ~5.94 Å. Interestingly, after NH_4^+ intercalation, the bond length of Sn-S becomes longer (2.55 Å) and that of W-S becomes shorter (2.38 Å). The interlayer distance of $Sn_{0.5}W_{0.5}S_2$ with intercalated NH_4^+ ions also becomes larger.

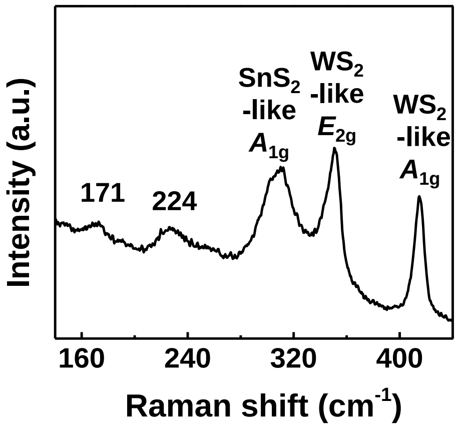

Supplementary Figure 11. Raman spectrum of as-prepared $Sn_{0.5}W_{0.5}S_2/SnS_2$ heterostructures.

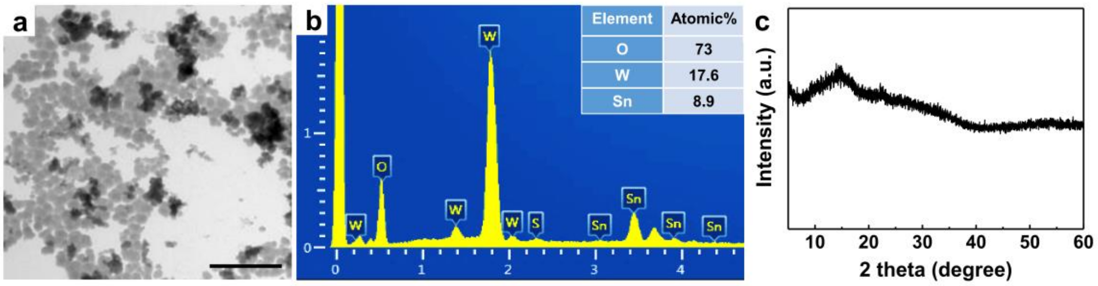

Supplementary Figure 12. (a) TEM image (scale bar, 500 nm), (b) EDX analysis and (c) XRD pattern of $Sn(HWO_4)_2{\cdot}nH_2O$ nanoparticles formed during pre-treatment of precursors in an 80 °C water bath before hydrothermal reaction. The XRD pattern in (c) shows no obvious peaks, suggesting that the $Sn(HWO_4)_2{\cdot}nH_2O$ nanoparticles were amorphous.

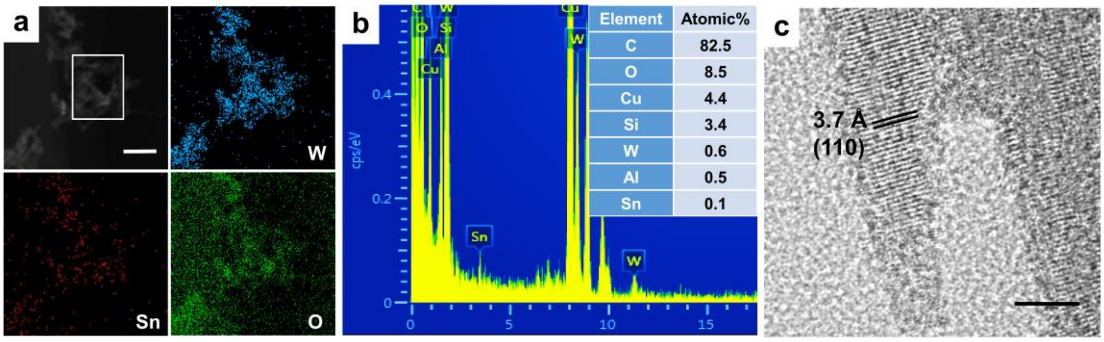

Supplementary Figure 13. (a) STEM image and EDS mapping of intermediate nanorods, confirming the presence of Sn and W elements (scale bar, 100 nm). (b) EDX analysis of the nanorods, where the position of the analyzed area is highlighted in the white rectangle in (a). The Sn:W ratio is about 0.1:0.6, suggesting that $Sn_{0.17}WO_3$ was obtained. (c) HRTEM image of the $Sn_{0.17}WO_3$ nanorods (scale bar, 5 nm). The measured lattice spacing of 3.7 Å can be assigned to the (110) planes of $Sn_{0.17}WO_3$ which show the same structure with previously reported $Sn_{0.23}WO_3$ (ICSD NO. 38043).

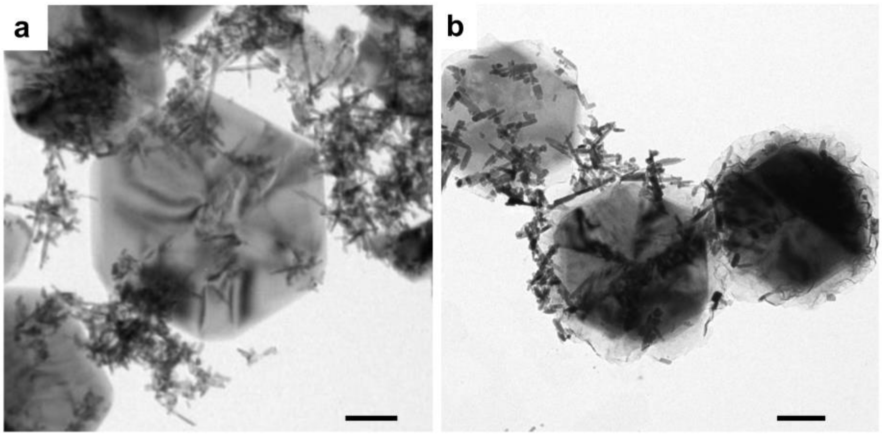

Supplementary Figure 14. TEM images of intermediate products obtained at reaction intervals of (a) 12 h and (b) 48 h, respectively. The scale bars, 200 nm.

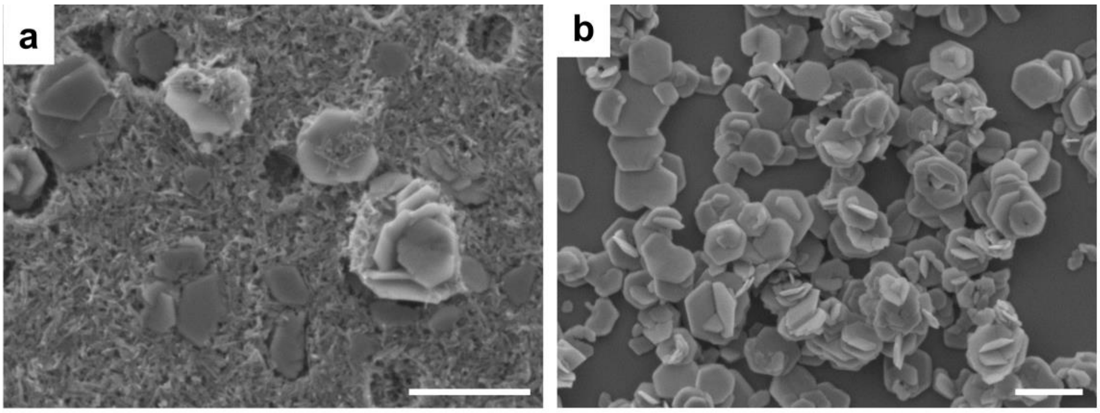

Supplementary Figure 15. SEM images of products synthesized at (a) 180 °C and (b) 200 °C for 60 h, respectively, without changing precursor concentrations (scale bars, 1 μm). It can be seen that $Sn_{0.17}WO_3$ nanorods could be prepared together with SnS_2 nanoplates at 180 °C, but were decomposed at 200 °C. $Sn_{0.5}W_{0.5}S_2$ nanosheets were not synthesized at 180 °C or 200 °C. This indicates that reaction at 220 °C with higher energy is needed for the synthesis of alloyed $Sn_{0.5}W_{0.5}S_2$. In addition, the quality of SnS_2 nanoplates prepared at 180 °C and 200 °C was not as good as those prepared at 220 °C.

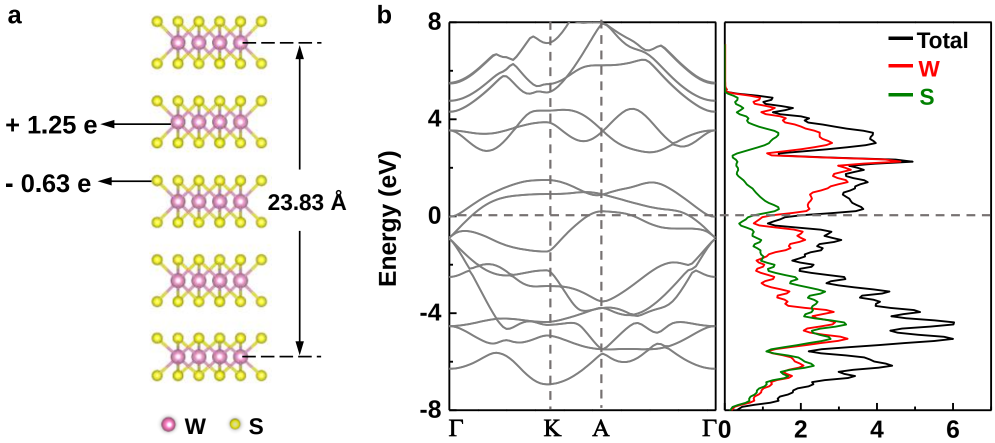

Supplementary Figure 16. (a) Optimized crystal structure with calculated Bader charges, (b) band structure and DOS of a 5-layer 1T-WS_2, demonstrating an intrinsic metallic behavior. The Fermi level is assigned at 0 eV.

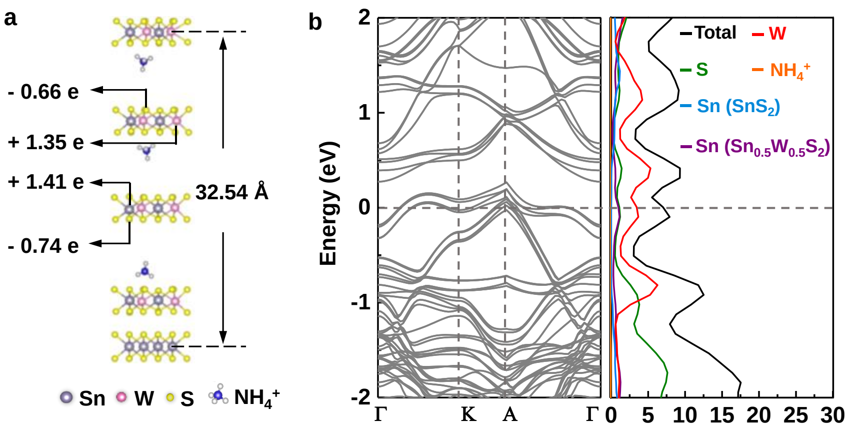

Supplementary Figure 17. (a) Optimized crystal structure with calculated Bader charges, (b) band structure and DOS of a four-layer distorted 1T-$Sn_{0.5}W_{0.5}S_2$ on a monolayer 1T-SnS_2 with intercalated NH_4^+ ions, showing an intrinsic metallic characteristic. The Fermi level is assigned at 0 eV.

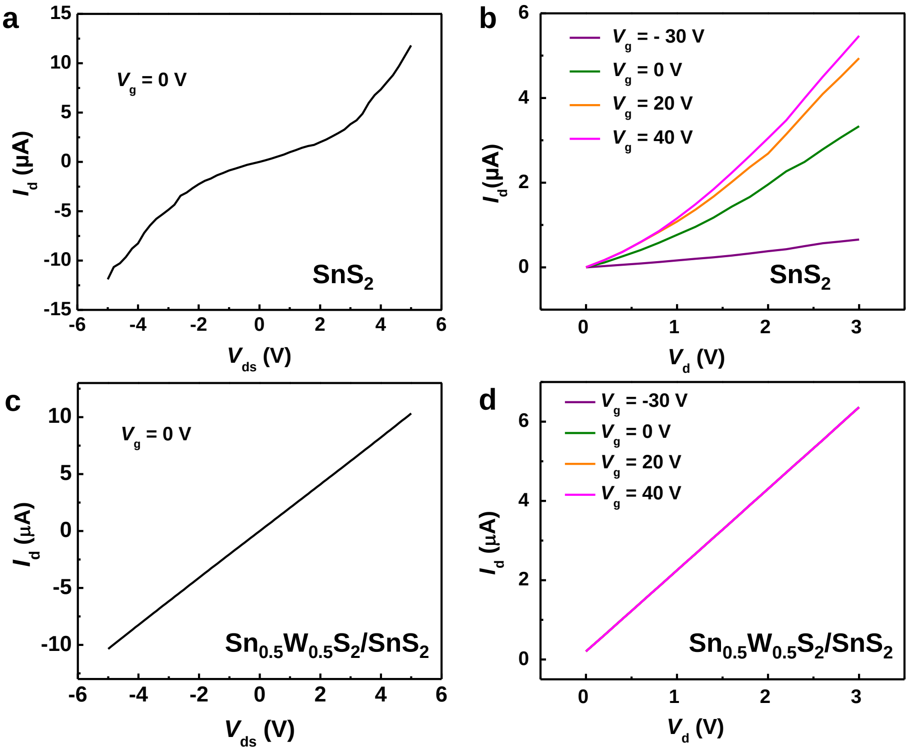

Supplementary Figure 18. Drain current (I_d) characteristics of back-gated TFTs based on (a) SnS_2 nanoplates and (c) $Sn_{0.5}W_{0.5}S_2/SnS_2$ heterostructures for drain-source voltages (V_{ds}) varied from -5 to 5 V at 0 V gate voltage (V_g). The I_d-V_{ds} curves of (b) SnS_2 nanoplates and (d) $Sn_{0.5}W_{0.5}S_2/SnS_2$ heterostructures at varied V_g from -30 to 40 V, measured in vacuum (5×10^{-5} Torr) at 77 K.

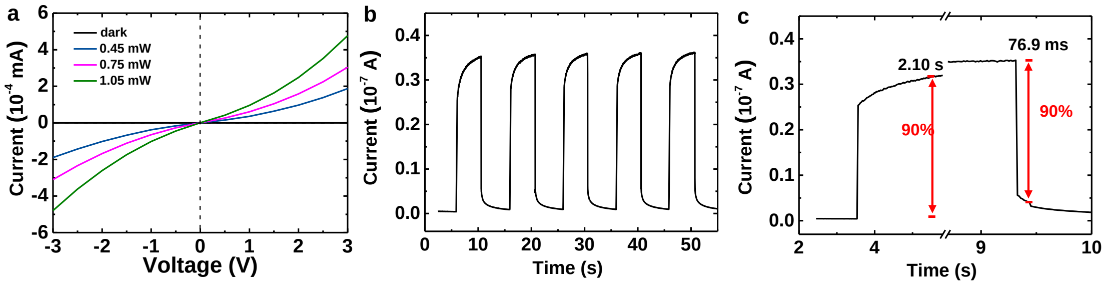

Supplementary Figure 19. (a) *I-V* curves at different light intensity, (b) temporal photocurrent response and (c) zoom-in view of the temporal photocurrent response of a thin film photodetector based on SnS_2 nanoplates.

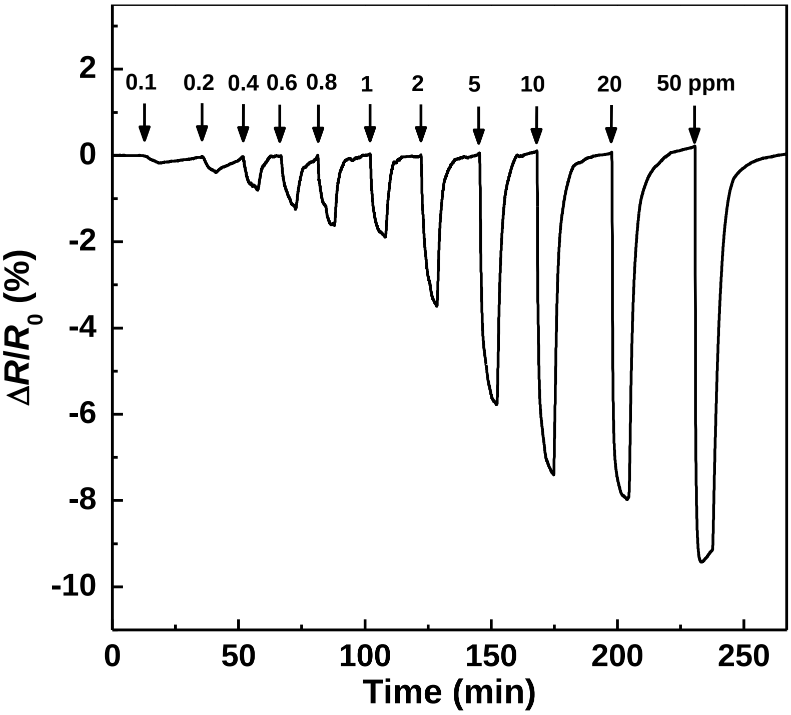

Supplementary Figure 20. Response-recover curve of a typical $Sn_{0.5}W_{0.5}S_2/SnS_2$-based sensor in response to acetone with different concentrations from 0.1 to 50 ppm.

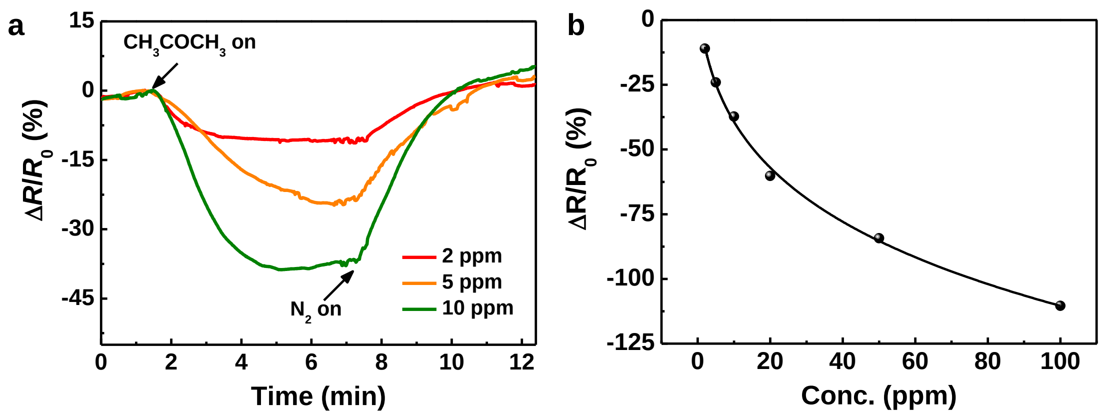

Supplementary Figure 21. (a) Response-recover curves of a typical SnS_2-based sensor in response to acetone with different concentrations from 2 to 10 ppm. (b) Normalized change of resistance of SnS_2 sensor at various acetone concentrations.

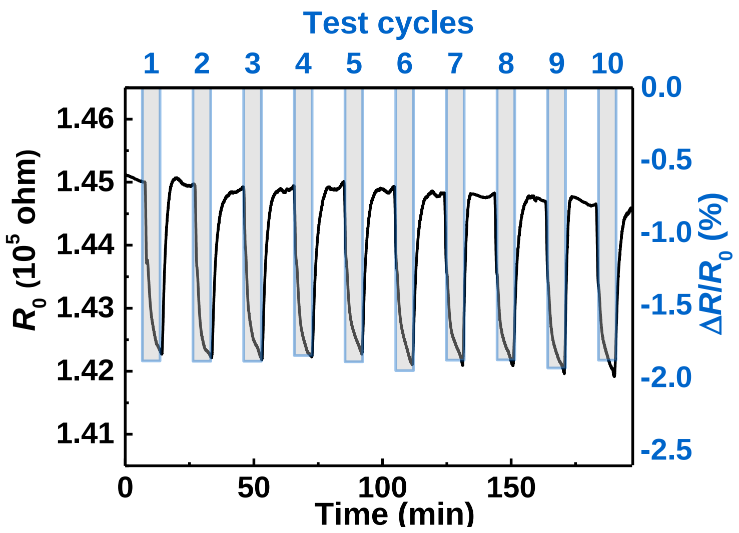

Supplementary Figure 22. Response-recover curve of $Sn_{0.5}W_{0.5}S_2/SnS_2$ heterostructure based sensor upon cyclic exposure to 1 ppm acetone. Overlayed bar chart: the corresponding responses for the tested 10 cylces.

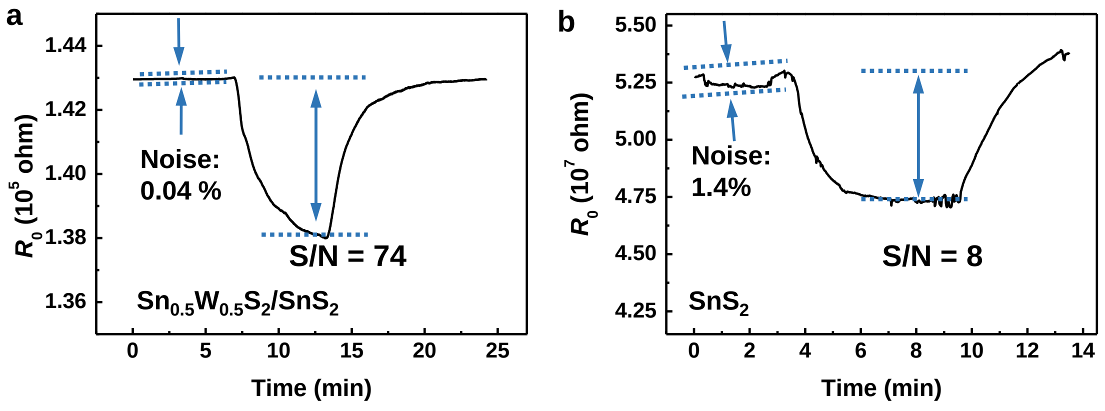

Supplementary Figure 23. The signal to noise ratios (S/N) of sensors based on (a) $Sn_{0.5}W_{0.5}S_2/SnS_2$ and (b) SnS_2 when responding to 2 ppm acetone.

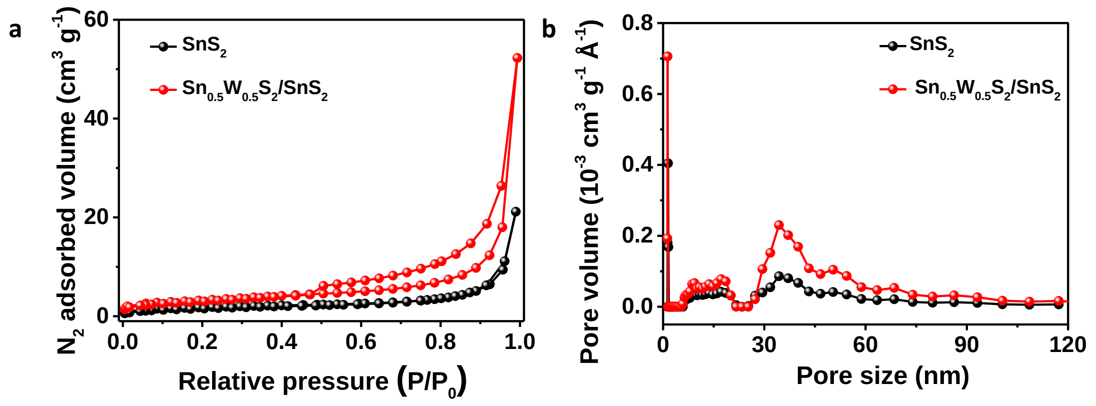

Supplementary Figure 24. (a) N_2 adsorption-desorption isotherms, and (b) DFT pore size distribution plots for SnS_2 nanoplates and $Sn_{0.5}W_{0.5}S_2/SnS_2$ heterostructures.

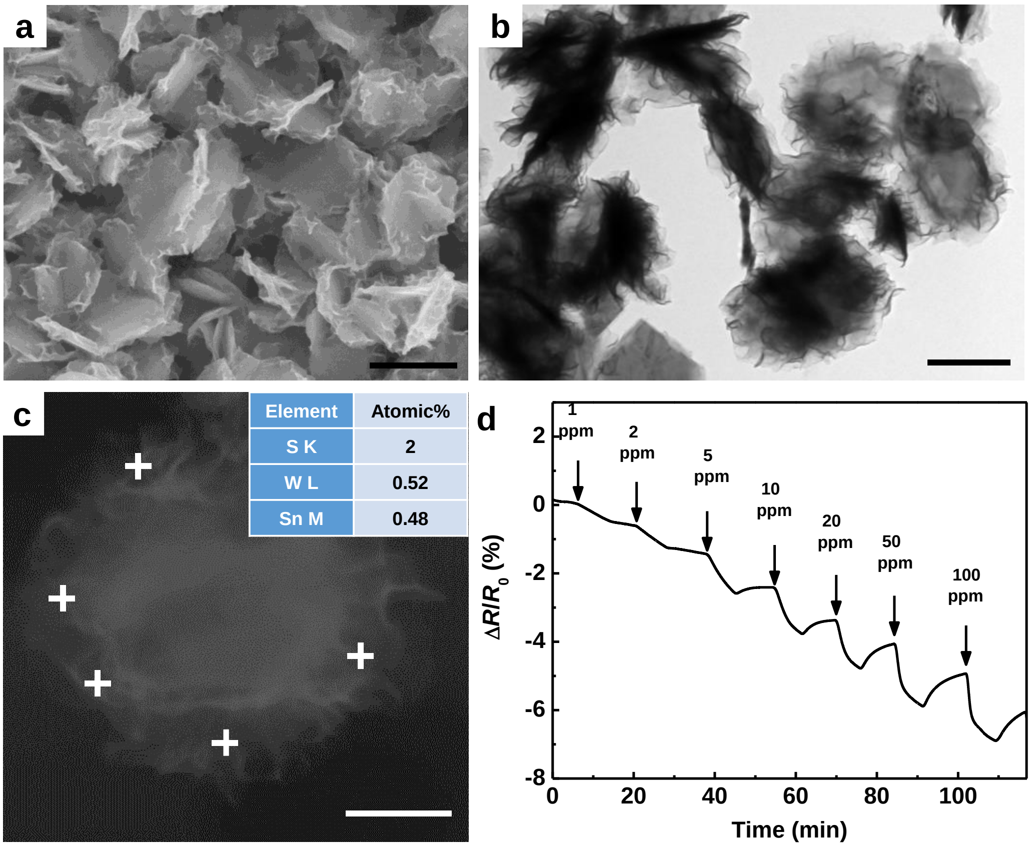

Supplementary Figure 25. (a) SEM image, (b) TEM image and (c) EDX spot analysis of $Sn_{0.5}W_{0.5}S_2/SnS_2$ heterostructures synthesized with precursors mixed at a Sn:W:S atomic ratio of 1:6:15. Increased amount of wrinkled $Sn_{0.5}W_{0.5}S_2$ nanosheets were found to encapsulate SnS_2 nanoplates. The scale bars in (a), (b) and (c) are 1 µm, 500 nm and 200 nm, respectively. (d) The response-recovery curve of the corresponding $Sn_{0.5}W_{0.5}S_2/SnS_2$ sensor upon exposure to acetone gas with increasing concentrations from 1 to 100 ppm.

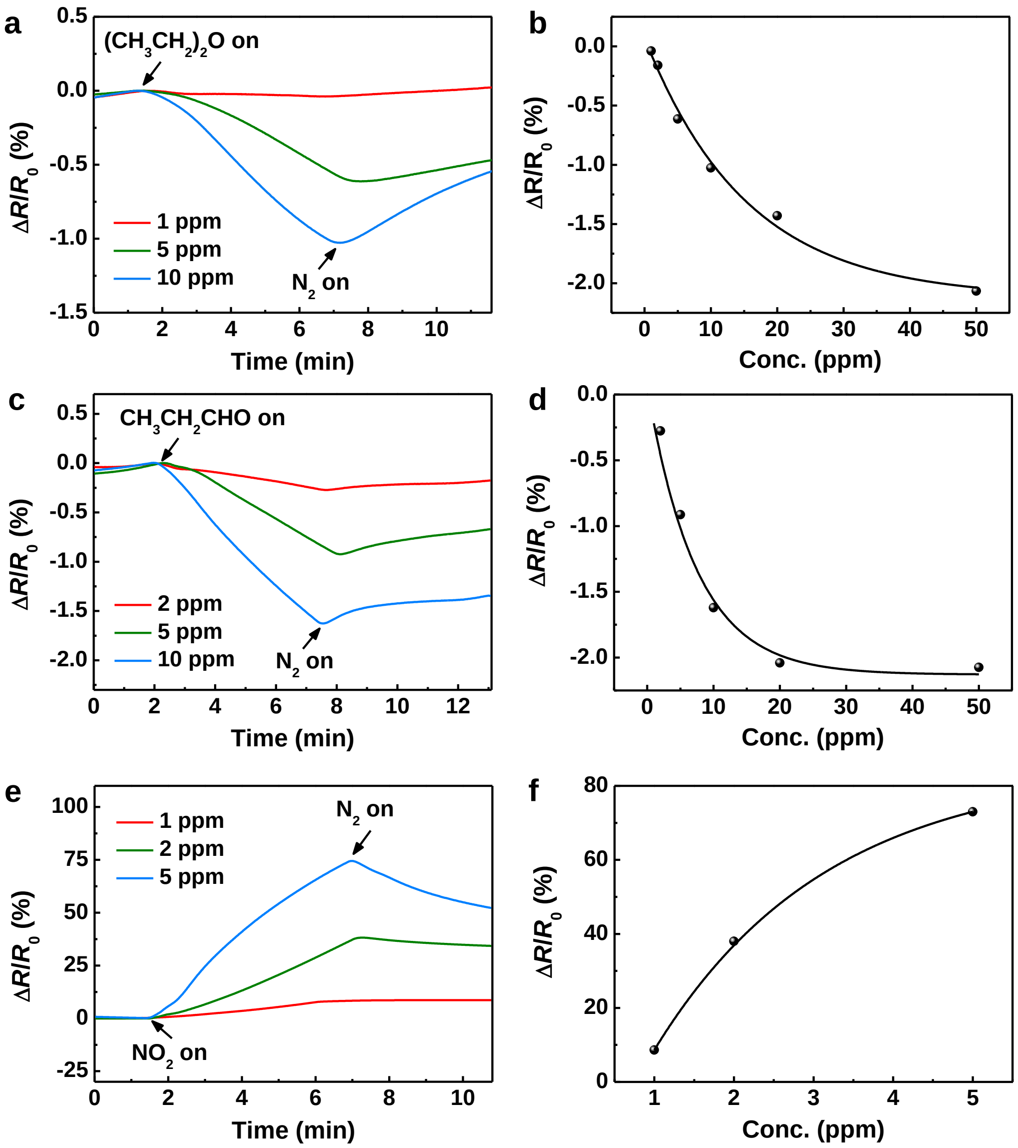

Supplementary Figure 26. Response-recover curves and normalized change of resistance of $Sn_{0.5}W_{0.5}S_2/SnS_2$ towards different gases including (a,b) diethyl ether, (c,d) propanal and (e,f) NO_2 at different concentrations.

Supplementary Tables

Supplementary Table 1. Optimized lattice parameters, bond lengths and work functions (Φ) of 1T-SnS_2, 1T-WS_2 and a four-layer $Sn_{0.5}W_{0.5}S_2$ on a monolayer 1T-SnS_2 without and with intercalated NH_4^+ ions.

System	*a* (Å)	*b* (Å)	*c* (Å)	*α* (°)	*β* (°)	*γ* (°)	Sn-S bond length (Å)	W-S bond length (Å)	*Φ* (eV)
SnS_2	3.65	3.65	23.60	90	90	120	2.58	-	5.27
WS_2	3.15	3.15	23.83	90	90	120	-	2.42	6.62
$Sn_{0.5}W_{0.5}S_2$	3.46*	3.46*	23.34	90	90	120	2.52	2.43	5.62
NH_4^+-intercalated $Sn_{0.5}W_{0.5}S_2$	3.46*	3.46*	32.54	90	90	120	2.55	2.38	2.81

* The experimental lattice constant (3.46 Å) was used in our calculations for the in-plane periodicity of the four-layer $Sn_{0.5}W_{0.5}S_2$ on a monolayer 1T-SnS_2 without and with NH_4^+-intercalated systems.

Supplementary Table 2. Concentrations of Sn and W ions measured by ICP-MS for solutions obtained at different reaction intervals.

Ions	5 h	12 h	24 h	48 h	60 h
Sn (μmol/L)	47.2	26.1	31.2	37.1	18.5
W (μmol/L)	566.2	877.9	1482.3	1283.2	1013.9

Supplementary Table 3. Comparison between our $Sn_{0.5}W_{0.5}S_2/SnS_2$-based sensor and other reported chemiresistive acetone gas sensors operating at room temperature.

Materials	Temperature (°C)	Sensor type	Minimum detectable concentration (ppm)	Response at 0.4 ppm (%)	Response at 0.2 ppm (%)	Ref.
$Sn_{0.5}W_{0.5}S_2/SnS_2$	25	Chemiresistor	0.1	0.60	0.37	This work
SnS_2	25	Chemiresistor	2.0	n.d.	n.d.	This work
MoS_2	25	Chemiresistor	1.0	n.d.	n.d.	4
SnS	25	Chemiresistor	10	n.d.	n.d.	5
Co_3O_4-ZnS	25	Chemiresistor	10	n.d.	n.d.	6
$Ti_3C_2T_x$	25	Chemiresistor	0.05	0.25*	0.18*	7
V_2O_5	25	Chemiresistor	0.9	n.d.	n.d.	8
WO_3-polyaniline	25	Chemiresistor	10	n.d.	n.d.	9
TiO_2	27	Chemiresistor	10	n.d.	n.d.	10
Pt-SnO_2	25	Chemiresistor	10	n.d.	n.d.	11
SnO_2-RGO	25	Chemiresistor	10	n.d.	n.d.	12
ZnO	25	Chemiresistor	2.0	n.d.	n.d.	13
C-ZrO_2	25	Chemiresistor	10	n.d.	n.d.	14
Ce-ZnO	24	Chemiresistor	1.0	n.d.	n.d.	15
InGa-ZnO	25	Chemiresistor	50	n.d.	n.d.	16
$La_{1-x}Sr_xCoO_3$	25	Chemiresistor	10	n.d.	n.d.	17
VO_2	25	Chemiresistor	5.0	n.d.	n.d.	18
$ZnWO_4$	23	Chemiresistor	100	n.d.	n.d.	19
TsCuPc/ZnO	35	Chemiresistor	50	n.d.	n.d.	20
Carbon onions	25	Chemiresistor	37	n.d.	n.d.	21

n.d. means non-detectable.

* denotes estimated value from the reported normalized response curve.

Supplementary References

1 Ou, J. Z. *et al.* Physisorption-based charge transfer in two-dimensional SnS_2 for selective and reversible NO_2 gas sensing. *ACS Nano* **9**, 10313-10323 (2015).

2 Liu, Q. *et al.* Gram-scale aqueous synthesis of stable few-layered 1T-MoS_2: applications for visible-light-driven photocatalytic hydrogen evolution. *Small* **11**, 5556-5564 (2015).

3 Eda, G. *et al.* Coherent atomic and electronic heterostructures of single-layer MoS_2. *ACS Nano* **6**, 7311-7317 (2012).

4 Kim, J. S., Yoo, H. W., Choi, H. O. & Jung, H. T. Tunable volatile organic compounds sensor by using thiolated ligand conjugation on MoS_2. *Nano Lett.* **14**, 5941-5947 (2014).

5 Afsar, M. F., Rafiq, M. A. & Tok, A. I. Y. Two-dimensional SnS nanoflakes: synthesis and application to acetone and alcohol sensors. *RSC Adv.* **7**, 21556-21566 (2017).

6 Park, S., Sun, G.-J., Kim, S., Lee, S. & Lee, C. UV-enhanced acetone gas sensing of Co_3O_4-decorated ZnS nanorod gas sensors. *Electron. Mater. Lett.* **11**, 572-579 (2015).

7 Kim, S. J. *et al.* Metallic $Ti_3C_2T_x$ MXene gas sensors with ultrahigh signal-to-noise ratio. *ACS Nano* **12**, 986-993 (2018).

8 Hakim, S. A., Liu, Y. L., Zakharova, G. S. & Chen, W. Synthesis of vanadium pentoxide nanoneedles by physical vapour deposition and their highly sensitive behavior towards acetone at room temperature. *RSC Adv.* **5**, 23489-23497 (2015).

9 Hicks, S. M. & Killard, A. J. Electrochemical impedance characterisation of tungsten trioxide–polyaniline nanocomposites for room temperature acetone sensing. *Sensors and Actuators B* **194**, 283-289 (2014).

10 Bhowmik, B., Hazra, A., Dutta, K. & Bhattacharyya, P. Repeatability and stability of room-temperature acetone sensor based on TiO_2 nanotubes: influence of stoichiometry variation. *IEEE Trans. Device Mater. Rel.* **14**, 961-967 (2014).

11 Shao, S. *et al.* Highly crystalline and ordered nanoporous SnO_2 thin films with enhanced acetone sensing property at room temperature. *J. Mater. Chem. C* **3**, 10819-10829 (2015).

12 Zhang, D., Liu, A., Chang, H. & Xia, B. Room-temperature high-performance acetone gas sensor based on hydrothermal synthesized SnO_2-reduced graphene oxide hybrid composite. *RSC Adv.* **5**, 3016-3022 (2015).

13 Muthukrishnan, K. *et al.* Studies on acetone sensing characteristics of ZnO thin film prepared by sol–gel dip coating. *J. Alloys. Compd.* **673**, 138-143 (2016).

14 Dankeaw, A., Poungchan, G., Panapoy, M. & Ksapabutr, B. In-situ one-step method for fabricating three-dimensional grass-like carbon-doped ZrO_2 films for room temperature alcohol and acetone sensors. *Sensors and Actuators B* **242**, 202-214 (2017).

15 Kulandaisamy, A. J. *et al.* Nanostructured Cerium-doped ZnO thin film – A breath sensor. *Ceram. Int.* **42**, 18289-18295 (2016).

16 Jaisutti, R., Kim, J., Park, S. K. & Kim, Y. H. Low-temperature photochemically activated amorphous indium-gallium-zinc oxide for highly stable room-temperature gas sensors. *ACS Appl. Mater. Interfaces* **8**, 20192-20199 (2016).

17 Liu, H. *et al.* Novel acetone sensing performance of $La_{1-x}Sr_xCoO_3$ nanoparticles at room temperature. *Sensors and Actuators B* **246**, 164-168 (2017).

18 Simo, A., Kaviyarasu, K., Mwakikunga, B., Mokwena, M. & Maaza, M. Room temperature volatile organic compound gas sensor based on vanadium oxide 1-dimension nanoparticles. *Ceram. Int.* **43**, 1347-1353 (2017).

19 Li, C. *et al.* Enhancement of gas-sensing abilities in p-type $ZnWO_4$ by local modification of Pt nanoparticles. *Anal. Chim. Acta* **927**, 107-116 (2016).

20 Bal, A. K. Room-temperature acetone sensing by sulfonated copper phthalocyanine (TsCuPc)-modified ZnO films. *J. Electron. Mater.* **44**, 144-151 (2014).

21 Dhonge, B. P., Motaung, D. E., Liu, C.-P., Li, Y.-C. & Mwakikunga, B. W. Nano-scale carbon onions produced by laser photolysis of toluene for detection of optical, humidity, acetone, methanol and ethanol stimuli. *Sensors and Actuators B* **215**, 30-38 (2015).
